# Signatures of Convergent Evolution and Natural Selection at the Alcohol Dehydrogenase Gene Region are Correlated with Agriculture in Ethnically Diverse Africans

**DOI:** 10.1093/molbev/msac183

**Published:** 2022-08-26

**Authors:** Michael A McQuillan, Alessia Ranciaro, Matthew E B Hansen, Shaohua Fan, William Beggs, Gurja Belay, Dawit Woldemeskel, Sarah A Tishkoff

**Affiliations:** Department of Genetics, University of Pennsylvania, Philadelphia, PA; Department of Genetics, University of Pennsylvania, Philadelphia, PA; Department of Genetics, University of Pennsylvania, Philadelphia, PA; Human Phenome Institute, School of Life Sciences, Fudan University, Shanghai, China; Department of Genetics, University of Pennsylvania, Philadelphia, PA; Department of Microbial Cellular and Molecular Biology, Addis Ababa University, Addis Ababa, Ethiopia; Department of Biology, Addis Ababa University, Addis Ababa, Ethiopia; Department of Genetics, University of Pennsylvania, Philadelphia, PA

**Keywords:** evolutionary biology, population genetics, natural selection, local adaptation, Africa, alcohol dehydrogenase

## Abstract

The alcohol dehydrogenase (ADH) family of genes encodes enzymes that catalyze the metabolism of ethanol into acetaldehyde. Nucleotide variation in ADH genes can affect the catalytic properties of these enzymes and is associated with a variety of traits, including alcoholism and cancer. Some ADH variants, including the ADH1B*48His (rs1229984) mutation in the *ADH1B* gene, reduce the risk of alcoholism and are under positive selection in multiple human populations. The advent of Neolithic agriculture and associated increase in fermented foods and beverages is hypothesized to have been a selective force acting on such variants. However, this hypothesis has not been tested in populations outside of Asia. Here, we use genome-wide selection scans to show that the ADH gene region is enriched for variants showing strong signals of positive selection in multiple Afroasiatic-speaking, agriculturalist populations from Ethiopia, and that this signal is unique among sub-Saharan Africans. We also observe strong selection signals at putatively functional variants in nearby lipid metabolism genes, which may influence evolutionary dynamics at the ADH region. Finally, we show that haplotypes carrying these selected variants were introduced into Northeast Africa from a West-Eurasian source within the last ∼2,000 years and experienced positive selection following admixture. These selection signals are not evident in nearby, genetically similar populations that practice hunting/gathering or pastoralist subsistence lifestyles, supporting the hypothesis that the emergence of agriculture shapes patterns of selection at ADH genes. Together, these results enhance our understanding of how adaptations to diverse environments and diets have influenced the African genomic landscape.

## Introduction

Modern humans arose in Africa ∼300,000 years ago, and African populations harbor the highest levels of genetic and phenotypic variation today (Rosenberg et al. 2002; Tishkoff et al. 2009; Hublin et al. 2017). Approximately 55,000–65,000 years ago, a relatively small number of people migrated out of Africa and subsequently populated the rest of the globe (Nielsen et al. 2017). During this dispersal out of Africa, humans interbred with now-extinct hominins, colonized novel environments, and encountered new pathogens. Each of these events induced unique selection pressures which have profoundly shaped the human genome (Scheinfeldt and Tishkoff 2013; Racimo et al. 2015; Fan et al. 2016). For example, many populations living at high altitude show signatures of positive natural selection at genes involved in the body’s response to hypoxia (Beall 2006; Scheinfeldt et al. 2012; Bigham and Lee 2014). Similarly, populations inhabiting regions with a history of endemic malaria display signatures of positive selection at many erythrocyte and immune-related genes (Kwiatkowski 2005; Hamid et al. 2021). Further, the transition from a hunting-gathering subsistence lifestyle to practicing agriculture and pastoralism over the last 10,000 years has resulted in drastic dietary changes in most human populations, which have induced further selection pressures (Luca et al. 2010). These dietary transitions have resulted in positive selection acting at genes involved in metabolism and energy production (e.g., lactose tolerance; Tishkoff et al. 2007; Ranciaro et al. 2014). While signatures of genomic adaptation to novel environments and diets have been extensively studied in many non-African populations, these patterns of natural selection within Africa have received less attention. However, due to Africa’s unique demographic history, studies of African genomic adaptation and natural selection are crucial in order to fully understand human evolutionary history, as well as the genetic basis of many complex traits and diseases (Sirugo et al. 2019). Here, we scanned the genomes of ethnically diverse Africans for signatures of positive natural selection and identified a particularly strong footprint of natural selection in a region flanking the alcohol dehydrogenase (ADH) genes in Ethiopian populations.

The ADH gene family consists of seven genes spanning an ∼350 kb region on chromosome 4. These genes and their protein products play a role in catalyzing the metabolism of ethanol into the toxic intermediate, acetaldehyde, which occurs primarily in the liver (reviewed in Edenberg 2007). Acetaldehyde is carcinogenic to humans and is responsible for many of the intoxicating effects of alcohol consumption (Guo and Jun 2010; Brooks and Zakhari 2014). Certain nucleotide variants in ADH genes alter enzyme kinematics and can impact the speed at which alcohol is converted into acetaldehyde (Birley et al. 2009; Hurley and Edenberg 2012). Variants that increase this conversion rate and therefore lead to local accumulation of acetaldehyde, strongly associate with a reduced risk of developing alcohol abuse disorders (Clarke et al. 2017). One well-known variant in this region is the nonsynonymous ADH1B*48His mutation (rs1229984) in the *ADH1B* gene, where a C > T mutation replaces an arginine with a histidine at residue 48 in the mature protein. This mutation results in a protein that catalyzes a significantly elevated ethanol-to-acetaldehyde conversion rate, which leads to local acetaldehyde accumulation in the blood. In addition, the mutation provides a protective effect against alcoholism and alcohol-related upper aerodigestive tract cancers (Bierut et al. 2012; Kang et al. 2014; Polimanti and Gelernter 2018).

Signatures of positive selection at ADH1B*48His have been identified in East Asians (Han et al. 2007; Peter et al. 2012; Wang et al. 2014), Southwest Asians (Gu et al. 2018), and Europeans (Galinsky et al. 2016), using *F*_ST_-based, haplotype-based, and principal component–based selection statistics. In East Asians, the ADH1B*48His variant was estimated to have arisen 7,000–10,000 years ago, which closely corresponds to the timing of rice domestication in China (Peng et al. 2010; Zuo et al. 2017). It was hypothesized that the associated increase in fermented foods and beverages during the Neolithic could have been the selective force that drove this variant to high frequency. However, a more recent analysis suggests that the East Asian haplogroup carrying the derived ADH1B*48His allele expanded more recently, around ∼2,800 years ago (Li, Gu, et al. 2011). Together, these studies suggest that ADH1B*48His has been the target of selection in multiple populations, but questions remain about the selective force responsible, the timing and strength of selection, and whether the emergence of agriculture has contributed to patterns of diversity at this locus. Also, it is unclear whether the ADH1B*48His allele alone is under strong selection, or if there are other variants on the same haplotype background contributing to this selection signal. These questions are complicated by research suggesting ADH1B*48His is involved in some alcohol-independent pathways and phenotypes, including cardiac and metabolic traits (Winnier et al. 2015; Polimanti et al. 2016). Finally, the ADH1B*48His allele has only been reported to be present in one sub-Saharan African population at appreciable frequency, the Ethiopian Jews (Gu et al. 2018), and the evolutionary dynamics affecting this and other ADH loci in Africa are generally unclear (but see Johnson and Voight 2018). We aim to address these shortcomings by studying patterns of variation, natural selection, and introgression at the ADH gene region in a set of ethnically diverse African populations, with a focus on populations in Northeast Africa, where the ADH1B*His allele is common.

Northeast Africans, and in particular Ethiopian populations, display extremely high levels of cultural, genetic, and phenotypic diversity. Over 70 languages are spoken in Ethiopia, comprising two broad language families—Afroasiatic, which is further broken into Cushitic, Semitic, and Omotic subfamilies, and Nilo-Saharan (www.ethnologue.com). Genetic structure is highly correlated with language in Ethiopia (Pagani et al. 2012). Further, a large component of the genetic ancestry of some Ethiopian populations, particularly the Cushitic and Semitic groups, originates from outside of Africa (Pickrell et al. 2014; Gallego et al. 2015; Pagani et al. 2015; Molinaro et al. 2019). While Ethiopians have been under-represented in human genetic studies, their rich ethnic, genetic, and linguistic diversity provides an ideal opportunity to disentangle the effects of genetic, cultural, and demographic factors in shaping patterns of variation and adaptation at the ADH gene region.

**Fig. 1.**
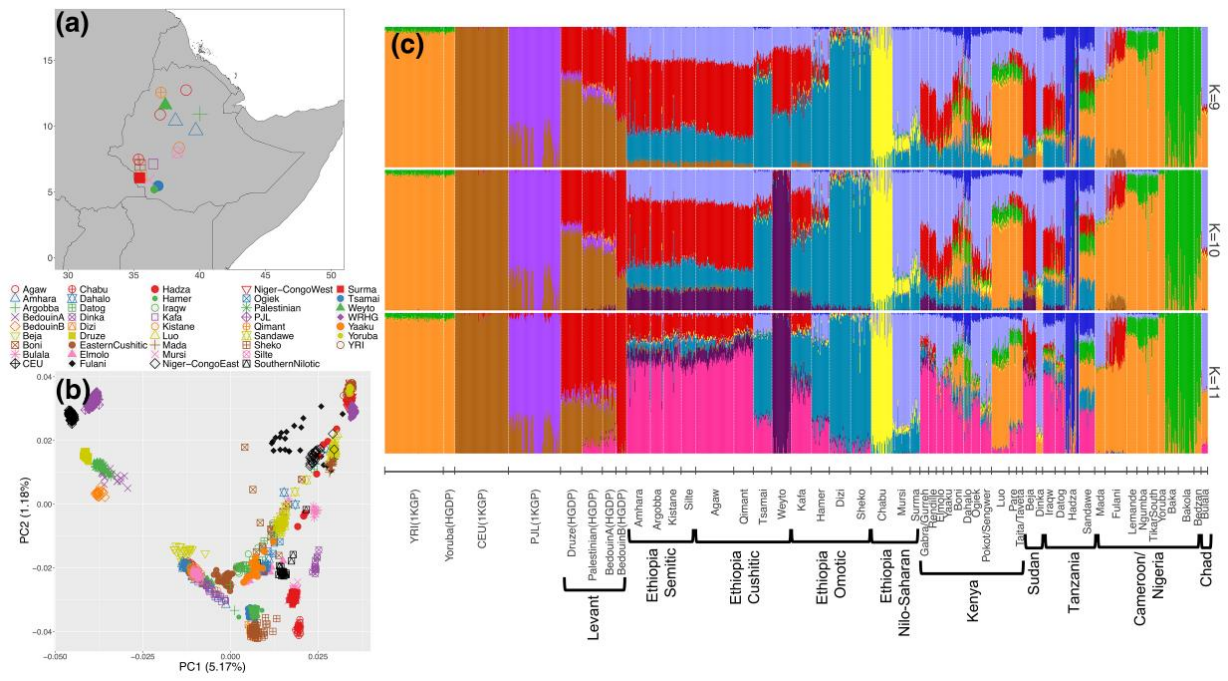
(*a*) Sampling locations of Ethiopian samples examined in this study for genome-wide patterns of positive selection. (*b*) Principal component analysis (PCA) of Ethiopian populations in combination with African and non-African populations. Ethiopian Afroasiatic Cushitic- and Semitic-speaking populations generally cluster together, while Omotic speakers form their own cluster. Nilo-Saharan speakers are distinct from other Ethiopians. (*c*) ADMIXTURE analysis of Ethiopians in combination with African and non-African populations. Results for *K* = 9 through 11 are shown. *K* = 11 had the smallest cross-validation error of all *K*-values. Language family is denoted below the plot for all Ethiopian populations. YRI, Yoruba from one thousand genomes (1KGP), CEU, Northern and Western European (1KGP), PJL, Punjabi (1KGP). For plotting purposes, some groupings contain multiple ethnicities.

In this study, we test for statistical signatures of recent positive selection in a genomic data set from 1,071 ethnically diverse Africans (fig. 1). These data represent much of the cultural, linguistic, and genetic diversity found within Africa, and include agriculturalist, pastoralist, agro-pastoralist, as well as hunter-gatherer groups. We identify a particularly strong signature of local adaptation in Ethiopia at the ADH gene region. We examine correlations between selection signals at this gene region and subsistence lifestyle and investigate patterns of introgression from non-African sources to better distinguish the roles of selection, gene flow, agriculture, and other cultural and environmental factors in shaping patterns of diversity at this locus.

## Results

### Inferring Population Structure in Ethnically Diverse Ethiopians

We performed ADMIXTURE (Alexander et al. 2009) and principal component analysis (PCA) to infer fine-scale population structure across ethnically diverse Ethiopian populations. To do this, we combined an Ethiopian genomic data set consisting of 541 individuals genotyped on the Illumina 5M array (∼4.2 million single-nucleotide polymorphisms [SNPs]; Crawford et al. 2017) with 530 African samples from Sudan, Kenya, Tanzania, Cameroon, and Chad genotyped on the Illumina 1M array (∼1 million SNPs; Scheinfeldt et al. 2019). We imputed missing genotypes in each data set separately using a reference panel of 180 diverse African whole-genome sequences (Fan et al., unpublished) and haplotypes from the 1,000 Genomes Project (1KGP; Auton et al. 2015) and merged the data sets together, resulting in a final data set of ∼20 million SNPs (see Materials and Methods). We merged this African data set (hereafter referred to as the “Africa-diversity” data set) with whole-genome sequence data for populations inhabiting the Levant from the Human Genome Diversity Project (HGDP; Bergström et al. 2020), and select global populations from the 1KGP. We performed ADMIXTURE analysis for *K*-values 4–15, and present results for *K* = 9–11 in figure 1. Broadly, ADMIXTURE and PCA results are consistent with each other. We find that samples from Ethiopia cluster into four main ancestral groups (fig. 1*b*and*c*). First, populations speaking Afroasiatic Semitic and Cushitic languages typically cluster together in PCA and share ancestry in ADMIXTURE, except for the Tsamai, a Cushitic-speaking group who are genetically more similar to some Omotic speakers. Also, the Weyto, a Cushitic-speaking hunter-gatherer group, become genetically distinct from other Cushitic/Semitic speakers at higher *K*-values in ADMIXTURE, although they cluster with other Semitic/Cushitic speakers in the PCA. Second, Omotic-speaking populations typically cluster together, with the exception of the Kafa, an Omotic-speaking group who show more similarity with some Semitic/Cushitic populations in both the PCA and ADMIXTURE analyses (fig. 1*b*and*c*). The third Ethiopian genetic cluster comprises a Nilo-Saharan-speaking group containing the Mursi and Surma ethnic groups, while the fourth genetic cluster comprises the Chabu hunter-gatherers, who are genetically differentiated from other Ethiopians, likely due to a recent bottleneck (Gopalan et al. 2022).

### Signatures of Positive Selection and Differentiation at the ADH Gene Region

To detect genome-wide signatures of positive selection, we first grouped the Ethiopian individuals (*n* = 541) into four major groups based on shared genetic ancestry as described above (fig. 1*b*and*c*; supplementary table S1, Supplementary Material online). Specifically, we combined all Semitic- and Cushitic-speaking individuals into an “Ethiopian Semitic/Cushitic” group, but excluded the Tsamai and Weyto ethnic groups, as the Tsamai cluster more closely to Omotic speakers and the Weyto become distinct at higher ADMIXTURE *K*-values (*K* = 10–11), likely due to genetic drift (fig. 1*b*and*c*). We combined all Omotic speakers into an “Ethiopian Omotic” grouping, but excluded the Kafa ethnic group, as they possess a large proportion of Semitic/Cushitic ancestry (fig. 1*c*). The Mursi and Surma ethnic groups comprise an “Ethiopian Nilo-Saharan” population, while the Ethiopian Chabu hunter-gatherers, who speak an uncharacterized language similar to Nilo-Saharan, comprise their own population. We further pruned the data set to omit individuals with first- and second-degree relationships. All other populations from the Africa-diversity data set which were included in the selection scans are as described in Scheinfeldt et al. (2019) and listed in supplementary table S1, Supplementary Material online, where we also list sample sizes after relatedness pruning.

**Fig. 2.**
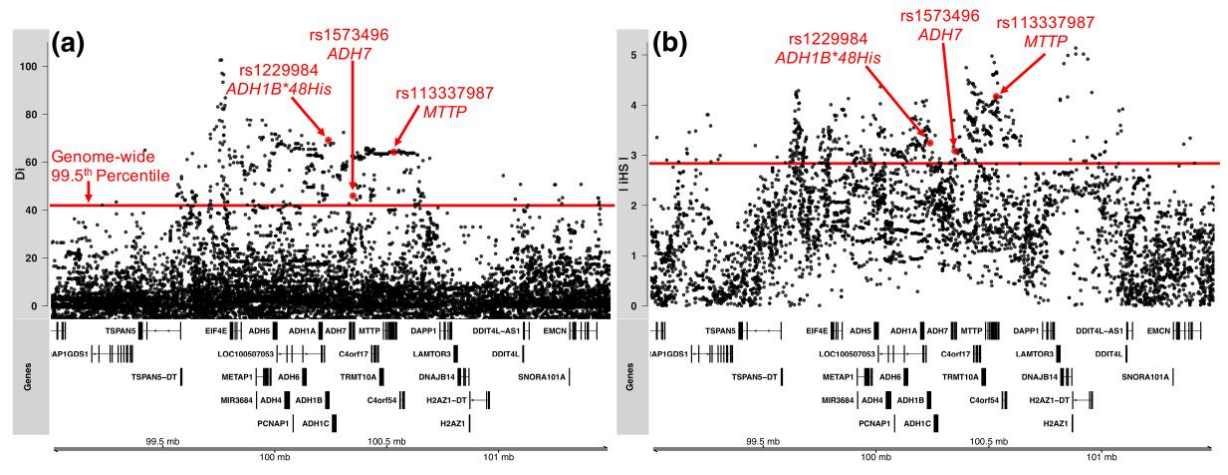
Signatures of positive selection across the ADH gene region in the pooled Ethiopian Semitic/Cushitic population (*n* = 220). (*a*) *D_i_* statistic and (*b*) iHS scores show a ∼1.5 Mb region of elevated scores across the ADH region. Red line denotes genome-wide empirical 99.5th percentile for each statistic, and nonsynonymous mutations discussed in the main text are highlighted with red arrows.

We tested for local adaptation in the Africa-diversity data set (supplementary table S1, Supplementary Material online) using the *D_i_* statistic developed by Akey et al. (2010). Briefly, the *D_i_* statistic is a function of standardized pairwise *F*_ST_ measurements between a focal population and all other populations in the data set and is useful for identifying variants that are highly differentiated in the focal population compared with all others. Large positive values of the *D_i_* statistic are, thus, indicative of adaptive signals specific to the focal population, where an allele’s frequency is highly differentiated compared with all other populations. From these scans, we observe a large region of significantly elevated *D_i_* scores, with many SNPs in the top 0.5% of the genome-wide distribution, centered around the ADH gene region on chromosome 4 in the pooled Ethiopian Semitic/Cushitic population (fig. 2*a*), but not in any other sub-Saharan African population (supplementary table S1, Supplementary Material online). This region of elevated *D_i_* stretches ∼1.5 Mb along chromosome 4, indicating that not only does this population possess many SNPs with highly divergent allele frequencies at this locus compared with the rest of sub-Saharan Africa, but that these SNPs may be located on unusually long haplotypes (fig. 2*a*).

In order to test for a signature of more recent positive selection in the ADH gene region, we calculated the integrated haplotype score (iHS), a haplotype-based statistic which measures the level of extended haplotype homozygosity (EHH) surrounding each site in the genome (Voight et al. 2006). This statistic compares levels of linkage disequilibrium (LD) surrounding a selectively favored allele relative to a nonselected allele at the same position and is sensitive to relatively recent selective sweeps. Again, we observe that the ADH gene region is enriched for SNPs possessing iHS scores well within the top 0.5% genome-wide in the pooled Ethiopian Semitic/Cushitic population, and that this region of elevated iHS stretches for ∼1.5 Mb (fig. 2*b*). These data indicate that the ADH gene region contains unusually long haplotypes relative to the rest of the genome in this population, suggesting positive selection has occurred in the recent past. Finally, we also calculated the iHS statistic genome-wide for all ethnic groupings individually in the Africa-diversity data set and find that this strong iHS signature of positive selection at the ADH gene region is unique to Ethiopian Afroasiatic-speaking populations (supplementary fig. S1, Supplementary Material online). To verify that these iHS and *D_i_* results were not impacted by cryptic relatedness in the data set, we repeated these analyses after pruning out individuals with third-degree relationships, and note the results do not change (supplementary fig. S2, Supplementary Material online).

### Candidate Functional Variants Under Selection

**Fig. 3.**
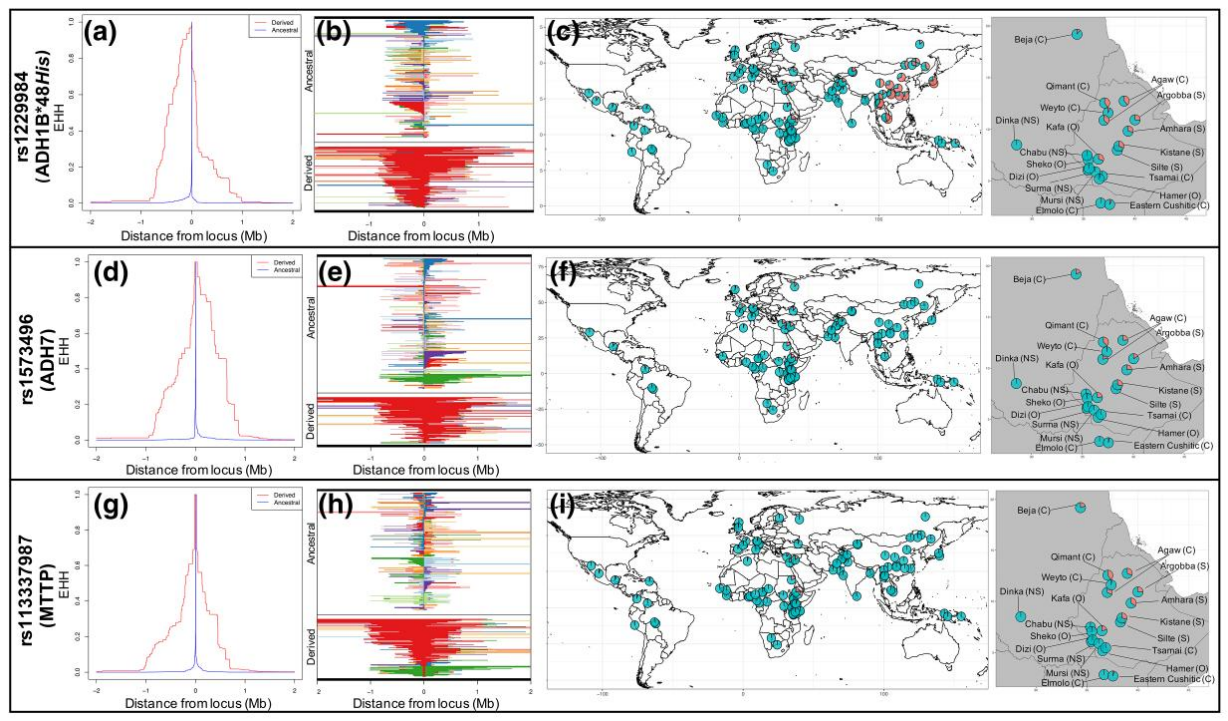
Patterns of extended haplotype homozygosity (EHH) in the pooled Ethiopian Semitic/Cushitic population and global allele frequency variation at nonsynonymous SNPs showing strong positive selection signals: ADH1B*48*His* (*a*–*c*), rs1573496 (*ADH7*) (*d*–*f*), and rs113337987 (*MTTP*) (*g*–*i*). For all three panels, the left-most figure shows the extent of haplotype homozygosity for the derived and ancestral alleles (*a*, *d*, *g*). In the next figure to the right, each row is a haplotype grouped by whether it carries the derived or ancestral allele. For each site along a haplotype, adjacent haplotypes of the same color are identical in sequence between that site and the central (selected) site (*b*, *e*, *h*). Right-most figures show global and Northeast Africa-specific allele frequencies (*c*, *f*, *i*). Abbreviations denote language family: S, Afroasiatic Semitic; C, Afroasiatic Cushitic; O, Afroasiatic Omotic; NS, Nilo-Saharan.

We next sought to characterize the putative functional variants displaying particularly strong selection signals in the pooled Ethiopian Semitic/Cushitic population by annotating all variants within a 2-Mb window centered on the ADH gene region. Within this window, we observe multiple exonic variants with high iHS and *D_i_* statistic scores. These exonic variants occur almost exclusively in ADH genes or in the microsomal triglyceride transfer protein (*MTTP*) gene ∼300 kb upstream of the ADH cluster (table 1), which plays an important role in lipid metabolism in the liver (see Discussion; Hussain et al. 2011). Here, we describe in detail three nonsynonymous variants that show both high iHS and *D_i_* scores: rs1229984 (ADH1B*48His), rs1573496 (*ADH7* gene), and rs113337987 (*MTTP* gene). The ADH1B*48His allele is at high frequency in the pooled Ethiopian Semitic/Cushitic population (33%), shows high iHS (iHS = 3.24, empirical *P* = 1.6 × 10^−3^) and *D_i_* (*D_i_* = 69.10, empirical *P* = 3.6 × 10^−4^) scores, and exhibits a signal of EHH extending >1 Mb up- and downstream of the derived allele (fig. 3*a*–*c*; table 1). In addition to ADH1B*48His, we also observe a strong selection signal at rs1573496, a nonsynonymous variant in the *ADH7* gene. In the pooled Ethiopian Semitic/Cushitic population, the derived allele frequency is high (26%), the variant shows high iHS and *D_i_* scores (iHS = 3.08, empirical *P* = 2.5 × 10^−3^; *D_i_* = 45.90, empirical *P* = 3.2 × 10^−3^), and the signal of EHH extends >1 Mb up- and downstream of the derived allele (fig. 3*d*–*f*; table 1). This variant is strongly associated with a reduced risk of developing alcohol-related upper aerodigestive tract cancers (Hashibe et al. 2008; McKay et al. 2011). Lastly, in the *MTTP* gene, we find a strong selection signal at the nonsynonymous variant rs113337987, which displays the highest iHS of all nonsynonymous variants in the region (iHS = 4.17, *P* = 1.2 × 10^−4^; fig. 3*g*–*i*; table 1). The derived allele frequency of this variant is 33% in the Ethiopian Semitic/Cushitic population and shows similarly broad patterns of EHH stretching >1 Mb flanking the allele.

**Table 1.** All exonic variants within 2 Mb of ADH1B*48His showing the strongest positive selection signals in the pooled Ethiopian Semitic/Cushitic population.

Chr	Position (hg19)	|iHS| (pval)	*D_i_* (pval)	Ancestral	Derived	Gene	Function	rsID	*R* ^2^ with ADH1B*48*His*	*D*′ with ADH1B*48*His*
4	100518283	4.5994 (0.00003)	39.9182 (0.00573)	C	T	MTTP	Synonymous SNV	rs17533489	0.414032	0.673279
**4**	**100532602**	**4.17267** (**0.00012)**	**65.137** (**0.00057)**	**G**	**A**	**MTTP**	**Nonsynonymous SNV**	**rs113337987**	**0**.**492046**	**0**.**716041**
4	100512849	3.87698 (0.00028)	64.137 (0.00056)	T	C	MTTP	Synonymous SNV	rs113557405	0.492046	0.716041
**4**	**100239319**	**3.24222** (**0.00162)**	**69.0986** (**0.00036)**	**C**	**T**	**ADH1B**	**Nonsynonymous SNV**	**rs1229984**	**-**	**-**
**4**	**100349669**	**3.08308** (**0.00252)**	**45.8996** (**0.00319)**	**C**	**G**	**ADH7**	**Nonsynonymous SNV**	**rs1573496**	**0**.**429619**	**0**.**789771**
4	100140306	3.04377 (0.00283)	17.8031 (0.05335)	T	A	ADH6	Nonsynonymous SNV	rs4699735	0.0600005	1
4	100263965	2.74795 (0.00651)	11.4911 (0.10432)	C	T	ADH1C	Nonsynonymous SNV	rs1693482	0.0643213	1
4	100516022	2.71836 (0.00705)	−9.16196 (0.85818)	G	C	MTTP	Nonsynonymous SNV	rs2306985	0.100461	0.72245
4	100510859	2.54494 (0.01135)	−9.21845 (0.86125)	C	T	MTTP	Synonymous SNV	rs991811	0.103183	0.728042
4	100485255	2.53289 (0.01173)	7.71301 (0.16029)	G	A	MTTP	Startloss	rs11944752	0.20251	0.652898
4	100341861	2.49383 (0.01304)	25.2181 (0.02502)	C	T	ADH7	Synonymous SNV	rs971074	0.442753	0.682467
4	100443720	2.48587 (0.01333)	1.69391 (0.3495)	G	A	C4orf17	Nonsynonymous SNV	rs13143848	0.0645508	0.625067
4	100504664	2.43168 (0.01534)	8.04044 (0.15411)	T	C	MTTP	Nonsynonymous SNV	rs3816873	0.20251	0.652898
4	99993833	2.33428 (0.01980)	−1.23324 (0.49645)	A	G	ADH5	Synonymous SNV	rs28730643	0.0657764	1
4	100266371	2.31467 (0.02083)	7.28774 (0.16868)	A	G	ADH1C	Synonymous SNV	rs1789915	0.0487944	1
4	100512412	2.19513 (0.02814)	25.6785 (0.0239)	T	C	MTTP	Synonymous SNV	rs982424	0.0500503	0.802629
4	100235053	2.16861 (0.03003)	0.54593 (0.40685)	G	A	ADH1B	Synonymous SNV	rs1789882	0.0501706	1
4	100266112	1.97141 (0.04823)	11.4911 (0.10432)	C	T	ADH1C	Synonymous SNV	rs1693425	0.0643213	1

Note.—Nonsynonymous mutations showing particularly strong selection signals that are examined in detail in main text are bolded.

**Fig. 4.**
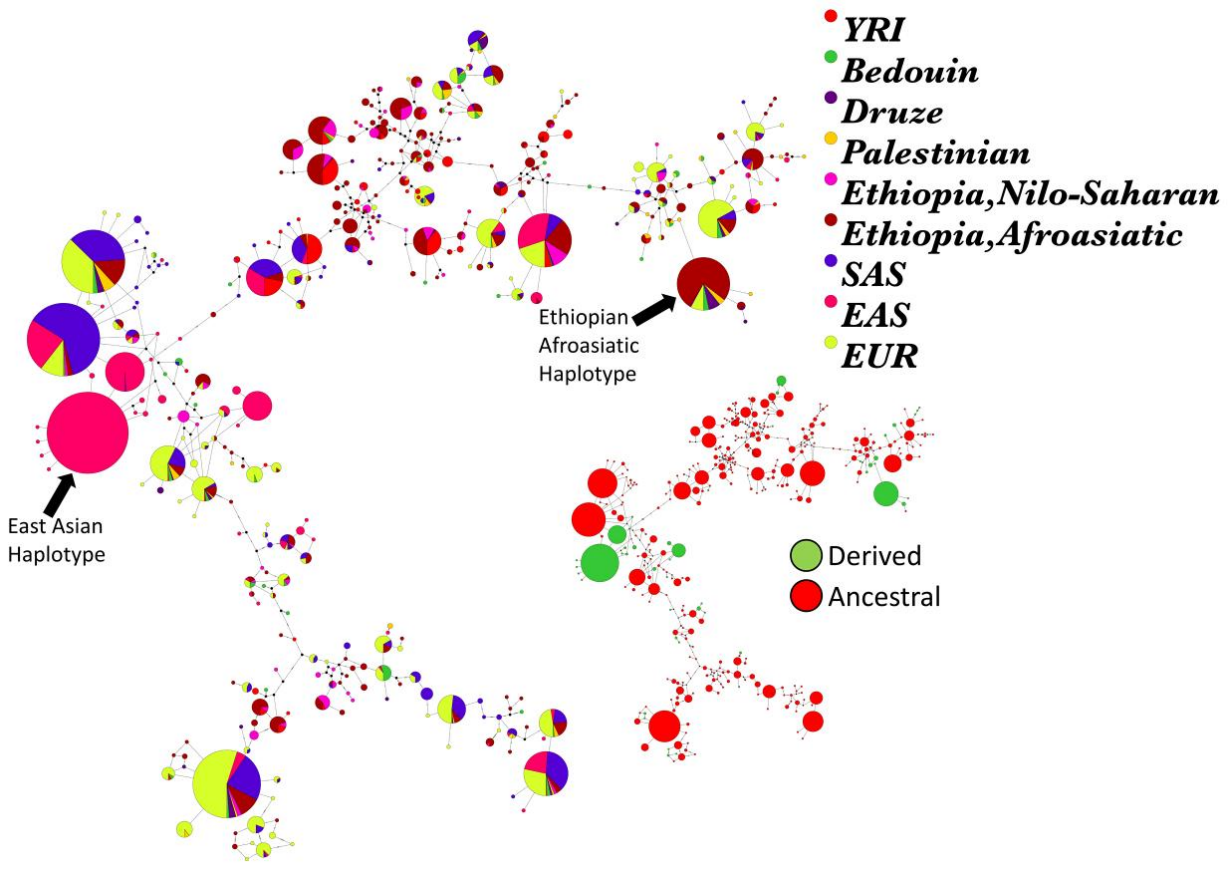
50kb median joining haplotype network around ADH1B*48His (108 SNPs). Larger network shows haplotypes colored by population, while smaller haplotype network is identical except that haplotypes are colored according to whether they carry the ancestral (red) or derived allele (green) at this locus. The Ethiopian Afroasiatic haplotype (dark red) carrying the derived allele is distinct from the East Asian (EAS, pink) haplotype. This Ethiopian haplotype is also identical to a haplotype found in the Levant (Druze, Palestinian, Bedouin) and Europe (EUR), suggesting identity by descent. Other population labels: YRI, Yoruba; SAS, South Asian.

We also examined phylogenetic relationships of haplotypes at the ADH region among global populations by constructing haplotype networks at these exonic SNPs with strong selection signatures. We first built a 50-kb haplotype network centered on rs1229984 (ADH1B*48His) (fig. 4). The most common haplotype containing the ADH1B*48His allele in Africans is identical to a haplotype found in Europeans from the 1KGP data set and populations from the Levant (i.e., Druze, Palestinian, and Bedouin who have the ADH1B*48His allele at 36%, 21% and 13% frequency, respectively), suggesting they are identical by descent. Furthermore, the ADH1B*48His haplotype in Europe, Africa, and the Levant is divergent from East Asian haplotypes carrying the derived ADH1B*48His allele, supporting previous claims of convergent evolution between Europe and Asia at this locus (Galinsky et al. 2016). We also constructed two separate 50 kb networks centered on rs1573496 (*ADH7*) and rs113337987 (*MTTP*), which also indicate that they are identical to haplotypes observed in populations from the Levant and Europe (supplementary figs. S3 and S4, Supplementary Material online). Next, we find that the haplotype carrying the three derived alleles at ADH1B*48His, rs1573496 (*ADH7*), and rs113337987 (*MTTP*), which are in moderately high, but not complete, LD with each other (*R*^2^ range 0.42–0.62; *D*′ range 0.72–0.93), is most common in Ethiopian Afroasiatic-speaking populations (18% frequency) compared with global populations. Again, this Ethiopian haplotype is identical to a haplotype found at low frequency in Europeans from the 1KGP data set (1%), and at moderate frequency in populations from the Levant (Druze = 13%, Palestinians = 6.5%, and Bedouin = 6.5%), suggesting identity by descent. The frequencies of the other haplotypes carrying combinations of derived and ancestral alleles at ADH1B*48His, rs1573496 (*ADH7*), and rs113337987 (*MTTP*) are broken down in supplementary fig. S5, Supplementary Material online.

There are several other notable nonsynonymous variants in the ADH gene region showing slightly diminished, but still significant, signals of positive selection in the pooled Ethiopian Semitic/Cushitic population (table 1). One variant in the *ADH1C* gene, rs1693482 (iHS = 2.75, *P* = 6.5 × 10^−3^) was shown to explain 8.4% of the variability in the metabolic rate of ethanol in a European cohort (Martínez et al. 2010). In the *MTTP* gene, the nonsynonymous SNP rs2306985 (iHS = 2.72, *P* = 7.1 × 10^−3^) was found to significantly associate with lower body mass index (BMI), waist circumference, and total cholesterol in a study of 7,582 German participants (Böhme et al. 2008). Finally, the nonsynonymous SNP rs3816873 in the *MTTP* gene (iHS = 2.43, *P* = 0.015) was found to significantly associate with lower levels of insulin, blood pressure, and prevalence of type II diabetes in a European population (Rubin et al. 2006). All of these variants have moderately high pairwise *D*′ LD values with ADH1B*48His in Ethiopia (range 0.65–1; table 1; supplementary figs. S5 and S6, Supplementary Material online), indicating they are often, but not always, located on the same haplotype background. We also note that the top *D_i_* SNP in the ADH region (rs10019726; fig. 2*a*) has high pairwise *D*′ LD values with ADH1B*48His, rs1573496 of *ADH7*, and rs113337987 of *MTTP* (0.96, 0.87, 0.85, respectively), though the *R*^2^ values are low (all pairwise < 0.32). However, given that this variant is not located in any known functional or regulatory region, it is unlikely to be the target of selection in this region.

Further, because many variants under selection are likely to fall in noncoding regions (Necsulea and Kaessmann 2014), we examined whether any variants in the ADH gene region displaying strong selection signals are predicted to have gene regulatory functions. To do this, we annotated all variants within 2 Mb of the ADH gene region using the Ensembl Variant Effect Predictor tool (McLaren et al. 2016), and examined variants labeled as “Regulatory Region Variants,” which are classified based on publicly available open chromatin and histone modification experimental data sets. We identify multiple regulatory candidates with elevated selection signals. First, rs150021439, an intergenic variant ∼700 kb upstream of the *ADH1B* gene, is predicted to be a regulatory variant, though the nearest gene is an uncharacterized noncoding RNA LOC256880. This variant has the third highest iHS of any variant in the 2 Mb region (iHS = 5.02, *P* = 1.0 × 10^−5^), is in moderate LD with ADH1B*48His (*R*^2^ = 0.24; *D*′ = 0.67), and was recently found to be significantly associated (*P* = 1.12 × 10^−22^) with the number of alcoholic drinks consumed per week in a large GWAS meta-analysis of primarily European subjects (Brazel et al. 2019). In addition, this variant is at higher frequency in the pooled Ethiopian Semitic/Cushitic population (21%) than any other global population in the African Diversity, HGDP, or 1KGP data sets (Druze = 11%; Palestinian/Bedouin = 7%; 0–5% in all other global populations). Another variant in intron 1 of the *ADH5* gene, rs1154402, also displays high iHS and *D_i_* values in the Ethiopian Semitic/Cushitic population (iHS = 3.41, *P* = 1.02 × 10^−3^; *D_i_* = 69.63, *P* = 3.4 × 10^−4^), is at high frequency (42.5%), and is a predicted regulatory variant within a promoter. A recent study found that this variant functions as an enhancer, and is significantly associated with expression levels of the *ADH1A* gene (Cui et al. 2018). This variant is also an expression quantitative trait locus (eQTL), significantly associated with the expression of *ADH1A*, *ADH4*, *ADH5*, *ADH1C*, and *ADH6* in multiple tissues according to the Genotype-Tissue Expression (GTEx) Project database (GTEx Consortium 2020). Further, this variant is significantly associated with risk of esophageal squamous-cell carcinoma in alcohol drinkers, but not in nondrinkers (Cui et al. 2018). Notably, this variant’s *D_i_* score is higher than any nonsynonymous variant in the ADH region and has a slightly higher iHS score than the nonsynonymous ADH1B*48His and rs1573496 *ADH7* variants. This variant is in high LD with ADH1B48*His in Ethiopia (*R*^2^ = 0.59; *D*′ = 0.93), making it challenging to distinguish the target(s) of selection. All other predicted regulatory variants in the ADH region with |iHS| > 2 are presented in Supplemental file 1, Supplementary Material online.

### Patterns of Variation at Variants Under Selection and Correlation with Subsistence

Next, we examined global as well as Northeast African-specific allele frequencies at the variants showing the strongest selection signals by combining our data with 76 additional populations from the 1KGP and the HGDP project data sets (Bergström et al. 2020). Within Africa, the derived alleles of the three nonsynonymous variants showing the strongest iHS and *D_i_* positive selection signals (ADH1B*48His, rs1573496 of *ADH7*, rs113337987 of *MTTP*) are only present at appreciable frequency (>5%) in Afroasiatic-speaking populations from Ethiopia, the Beja population from Sudan (contains Baniamer and Hadandawa ethnic groups), and the Eastern Cushitic-speaking population from Kenya (contains Gabrah, Gurreh, Rendille ethnic groups; fig. 3). Among the sampled Ethiopian populations, the presence of ADH1B*48His is confined to northern and central Ethiopia, primarily in Cushitic- and Semitic-speaking populations (fig. 3*c*). Interestingly, among the Cushitic- and Semitic-speaking populations possessing the ADH1B*48His allele in Ethiopia, the Weyto (a hunter-gatherer group), have the lowest allele frequency (7%). In contrast, in all other agriculturalist Cushitic- and Semitic-speaking ethnic groups in Ethiopia which possess the allele, the frequency ranges from 27% to 40%. Among the sampled Omotic-speaking populations in Ethiopia, only the Hamer and the Kafa possess the ADH1B*48His allele, at 3% and 30% frequency, respectively. The relatively high frequency of this allele in the Kafa may be explained by the genetic ancestry shared between the Kafa and other Cushitic and Semitic speakers (ADMIXTURE and PCA results; fig. 1*b*and*c*). This frequency difference between the Hamer and the Kafa may also be explained by differences in subsistence strategy. The Kafa are an agriculturalist group and have a high ADH1B*48His allele frequency, whereas the Hamer are a pastoralist group and have a low frequency of the allele. The ADH1B*48His allele is not present in any Nilo-Saharan-speaking group in Ethiopia. Outside of Ethiopia, ADH1B*48His is present at 10% frequency in the Beja from Sudan and at 7% in the Kenyan Eastern Cushitic population (fig. 3*c*). We observe a very similar allele frequency distribution in Northeast Africa at rs1573496, the nonsynonymous *ADH7* gene variant. This variant, however, is largely absent from East Asia and is only at high frequency in Ethiopia and the middle East (fig. 3*f*). Finally, the nonsynonymous *MTTP* gene variant rs113337987 shows a very similar frequency distribution within Northeast Africa as the other two variants but is unique in that the derived allele is only found at high frequency in Ethiopian Semitic- and Cushitic-speaking populations, and is absent or at low frequency in all other global populations (fig. 3*i*).

**Fig. 5.**
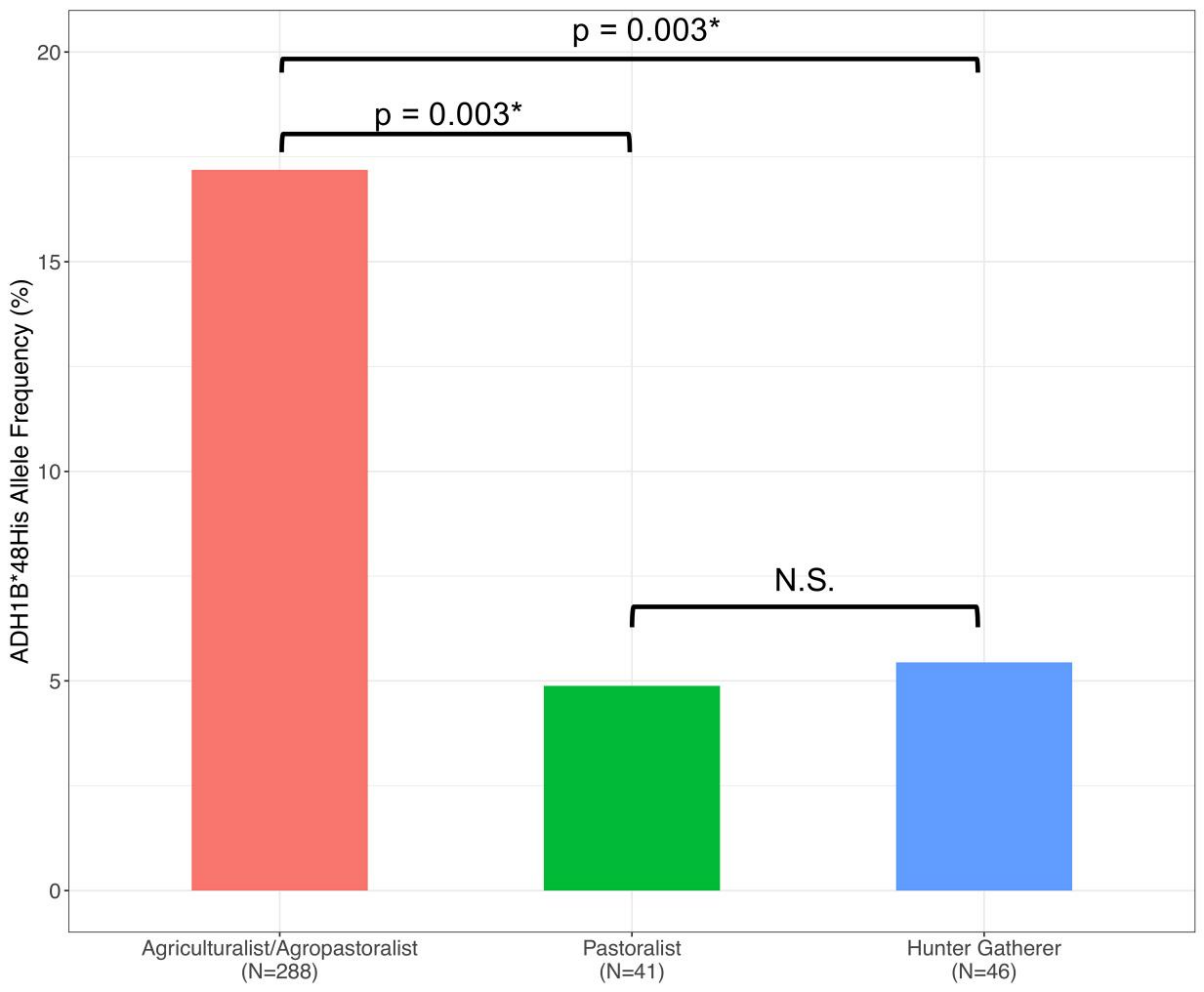
ADH1B*48His allele frequency in genetically similar Northeast African Afroasiatic-speaking individuals grouped by subsistence strategy. Analysis includes a subset of Afroasiatic speakers who are genetically similar based on ADMIXTURE results at *K* = 9. ADH1B*48His has a significantly higher allele frequency in agriculturalists compared with pastoralists and hunter-gatherers, based on fisher’s exact tests.

Patterns of variation and signatures of positive selection at these ADH loci are correlated with subsistence, as all populations showing these strong selection signals practice agriculture. For example, although the Ethiopian Weyto hunter-gatherers possess the ADH1B*48His allele at 7% frequency, we find no signal of positive selection at the ADH gene region in this population (supplementary fig. S7, Supplementary Material online). This is despite the fact that the Weyto are genetically similar to other Ethiopian Cushitic- and Semitic-speaking agriculturalist groups who show strong selection signals at this locus, based on PCA and ADMIXTURE analyses (fig. 1*b*and*c*). Similarly, the ADH1B*48His allele is unlikely to be under strong positive selection in the Boni or Yaaku ethnic groups, two Cushitic-speaking hunter-gatherer populations from Kenya who possess the allele at 2% and 3% frequency, respectively. We also do not find a signal of positive selection at the ADH gene region in the Kenyan Eastern Cushitic population, a pastoralist group who possess the ADH1B*48His allele at 7% frequency, even though this population is genetically very similar to Ethiopian Semitic/Cushitic-speaking agriculturalists (fig. 1*b*and*c*). We do find a signal of positive selection at ADH1B*48His in the pastoralist Beja population from Sudan (ADH1B*48His allele frequency = 10%, iHS = 2.15, *P* = 0.0304; *D_i_* not significant), although this signal does not extend across the ADH region on chromosome 4, and is much weaker than what we observe in Ethiopian Agriculturalists (supplementary figs. S1 and S8, Supplementary Material online). Finally, we examined the difference in ADH1B*48His allele frequency between genetically similar Afroasiatic-speaking agriculturalists, pastoralists, and hunter-gatherers. To do this, we used ADMIXTURE results at *K* = 9 (fig. 1) to identify Afroasiatic-speaking individuals with similar genetic ancestry and grouped them into three groups based on subsistence (agriculturalist, pastoralist, or hunter-gatherer). We only included individuals with the “dark teal” ancestry component ≥20%, as this component is common in East African Afroasiatic speakers and distinguishes them from the Levant. We find that the frequency of the ADH1B*48His allele is significantly higher in agriculturalists than in pastoralists or hunter-gatherers (17.2% allele frequency in agriculturalists vs. 4.9% in pastoralists and 5.4% in hunter-gatherers; fig. 5). Together, these results support the notion that the ADH gene region has only experienced positive selection in Afroasiatic-speaking agriculturalist populations, but not in Afroasiatic-speaking hunter-gatherers or pastoralists, while accounting for genetic ancestry.

### Patterns of Local Ancestry and Introgression at the ADH Gene Region

**Fig. 6.**
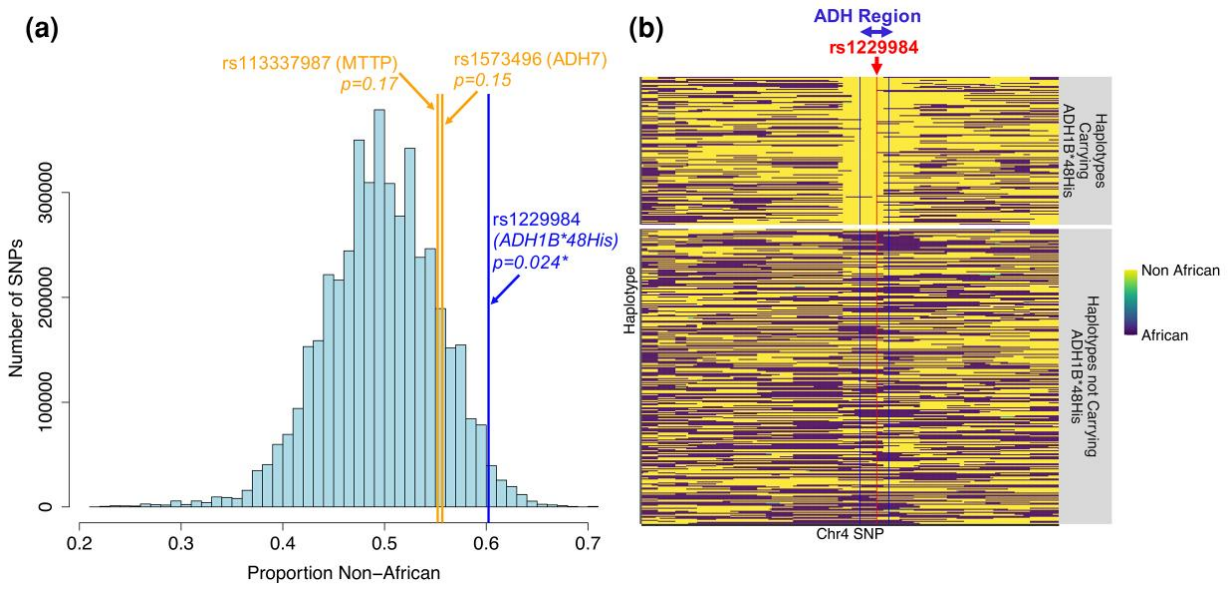
Patterns of local ancestry at the alcohol dehydrogenase gene region in the pooled Ethiopian Semitic/Cushitic population. (*a*) Histogram showing the proportion of haplotypes inferred to be “non-African” at each SNP genome-wide. Compared with the rest of the genome, the rs1229984 site (which contains the ADH1B*48His allele, blue vertical line) is at the tail-end of the empirical distribution for the proportion of haplotypes in the population inferred to be non-African, suggesting positive selection post-admixture. In contrast, the rs1573496 (*ADH7*) and rs113337987 (*MTTP*) sites (orange vertical lines) are not at the tail-end of the empirical distribution. (*b*) RFmix local ancestry results. Each row is a haplotype, and the color of the haplotype represents African (purple) or non-African (yellow) ancestry inferred at each position along the haplotype. Haplotypes carrying the ADH1B*48His allele are almost entirely inferred to be non-African in origin at the ADH region.

Because the Ethiopian Afroasiatic haplotypes carrying these positively selected variants are identical to haplotypes found in the Levant (fig. 4, supplementary figs. S3 and S4, Supplementary Material online), we tested whether these Ethiopian haplotypes are the result of introgression from a non-African source. To do this, we performed local ancestry inference using the program RFmix (v2; Maples et al. 2013). Given a focal population of interest and a panel of two or more ancestral reference populations, this methodology estimates the ancestral population of origin at each site across the genome, and outputs these estimates as tracks of local ancestry. We employed this framework to finely estimate local ancestry patterns in the genomes of individuals from the pooled Semitic/Cushitic Ethiopian population. We used the Yoruba from Nigeria as a West African reference, and the Dizi, an Omotic-speaking group from Ethiopia and a population we find to have low amounts of non-African admixture, as an East African reference (fig. 1*b*and*c*). For the non-African reference, we used the Druze from Israel, following previous studies showing that the non-African component of many Ethiopian genomes is most closely related to populations inhabiting the Levant (Pagani et al. 2012). From this analysis, we find that the proportion of haplotypes in the Ethiopian Semitic/Cushitic population inferred to be non-African at the rs1229984 site (ADH1B*48His) is higher than the genome-wide average when considering all SNPs as well as SNPs with a similar minor allele frequency in the focal population, and is at the tail-end of the empirical distribution (empirical *P* = 0.024; fig. 6*a*; supplementary fig. S9, Supplementary Material online). In addition, haplotypes carrying the ADH1B*48His allele in the pooled Ethiopian Semitic/Cushitic population are almost exclusively inferred to be of non-African origin in the region surrounding the *ADH1B* gene (fig. 6*b*). These results suggest that the haplotype carrying this variant likely introgressed from a non-African source, and that it likely was a target of positive selection after being introduced into Ethiopia. Haplotypes carrying the derived alleles at nonsynonymous SNPs rs1573496 (*ADH7*) and rs113337987 (*MTTP*) are also almost exclusively inferred to be of non-African origin, though these sites are not significantly enriched for non-African ancestry at a genome-wide level (fig. 6*a*, supplementary figs. S10 and S11, Supplementary Material online).

Finally, we looked to see if the ADH region, and the ADH1B*48His allele in particular, shows a signature of positive selection in the Druze population, which we presume to be a population closely related to the introgression source population. We find that ADH1B*48His does display a signal of EHH compared with the ancestral allele, but that this EHH signal decays more rapidly in the Druze than in the pooled Ethiopian Semitic/Cushitic population. Specifically, the EHH signal for ADH1B*48His in the Druze decays to below 0.05 by ∼500 kb upstream of the mutation, and ∼100 kb downstream. In contrast, the EHH signal for ADH1B*48His in the Ethiopian Semitic/Cushitic population does not decay to <0.05 until ∼700 kb upstream and ∼750 kb downstream of the mutation (supplementary fig. S12, Supplementary Material online). We also find that genome-wide calculated iHS values for ADH1B*48His, rs1573496 (*ADH7*), and rs113337987 (*MTTP*) are lower in the Druze than in the Ethiopian Semitic/Cushitic population (|iHS| = 2.07, 2.88, 2.02 for the three loci in the Druze, compared with 3.24, 3.08, and 4.17 in the Ethiopian Semitic/Cushitic group; supplementary table S2, Supplementary Material online). However, these iHS values in the Druze are within the top 5% of the empirical distribution of genome-wide iHS scores. These results suggest either weaker selection in the Druze than in Ethiopia or that positive selection in the Druze began at an earlier time period, and recombination has since broken down the haplotype. Selection at this locus in the Levant could be consistent with agriculture as the selective force, as the Levant was home to some of the world’s earliest agriculturalists (Kuijt and Goring-Morris 2002).

### Timing of Introgression at the ADH Gene Region and Strength of Selection

In order to determine how long ago this introgression occurred from outside of Africa into Ethiopia, we utilized the program *ALDER*, which measures the decay of admixture-induced LD as a function of genetic distance to date admixture events (Loh et al. 2013). We analyzed each of the Northeast African Afroasiatic-speaking ethnic groups as focal populations and used the Druze as a non-African reference and the Yoruba as an African reference. In these analyses, *ALDER* treats the focal population as an admixed population derived from an admixture event between the two reference populations and estimates the timing of this admixture. We observe admixture timings ranging from 57 to 93 generations ago (1713-2793 years ago) for Afroasiatic Cushitic- and Semitic-speaking populations from Ethiopia, depending on the focal population analyzed (table 2). We also considered the pooled Ethiopian Semitic/Cushitic population as a focal population in this analysis and estimate an admixture timing of 68.39 (±2.53) generations, or 2,052 (±75.9) years ago (table 2).

**Table 2.** *ALDER* results for estimates of admixture timing for Northeast African Afroasiatic-speaking populations, modeled as two-way admixture events between the Yoruba (YRI, Nigeria) and the Druze (Israel).

Population (*N*)	Country	Language	Yoruba–Druze Admixture Date: Generations Ago (± SE)	Yoruba–Druze Admixture Date: Years Ago (± SE)
Pooled Afroasiatic Semitic/Cushitic (220)	Ethiopia	Afroasiatic Semitic/Cushitic	68.39 (±2.53)	2,052 (±75.9)
Agaw (70)	Ethiopia	Afroasiatic Cushitic	71.81 (±6.03)	2,154 (±180.9)
Amhara (44)	Ethiopia	Afroasiatic Semitic	65.80 (±6.91)	1,974 (±207.3)
Argobba (24)	Ethiopia	Afroasiatic Semitic	70.40 (±6.74)	2,112 (±202.2)
Dizi (39)	Ethiopia	Afroasiatic Omotic	103.44 (±11.12)	3,103 (±336)
Hamer (33)	Ethiopia	Afroasiatic Omotic	99.28 (±13.28)	2,978 (±398.4)
Kafa (38)	Ethiopia	Afroasiatic Omotic	32.71 (±3.39)	981.3 (±101.7)
Kistane (33)	Ethiopia	Afroasiatic Semitic	71.86 (±7.16)	2,156 (±214.8)
Qimant (36)	Ethiopia	Afroasiatic Cushitic	93.09 (±7.65)	2,793 (±229.5)
Sheko (38)	Ethiopia	Afroasiatic Omotic	56.61 (±10.14)	1,698 (±304.2)
Silte (27)	Ethiopia	Afroasiatic Semitic	76.41 (±4.96)	2,292 (±148.8)
Weyto (35)	Ethiopia	Afroasiatic Cushitic	57.10 (±3.67)	1,713 (±110.1)
Boni (21)	Kenya	Afroasiatic Cushitic	9.66 (±2.60)	289.8 (±78)
Dahalo (14)	Kenya	Afroasiatic Cushitic	24.63 (±7.73)	738.9 (±231.9)
Eastern Cushitic (30)	Kenya	Afroasiatic Cushitic	48.50 (±7.25)	1,455 (±217.5)
Beja (25)	Sudan	Afroasiatic Cushitic	50.79 (±4.87)	1,523 (±146.1)

Note.—Admixture timing estimates are shown only for populations where the *ALDER* test of admixture passed. Admixture timing in years assumes a 30-year generation time.

We next calculated a selection coefficient for ADH1B*48His post-admixture by utilizing our local ancestry and admixture timing results. The estimated selection coefficient for the allele post-admixture in the pooled Ethiopian Semitic/Cushitic group is *s* = 1.12% (0.99–1.44%, likelihood ratio >1/100 interval [LR_100_]; see Materials and Methods). This assumes 68 generations since admixture, an initial postadmixture proportion of introduced non-African haplotypes of 50% (the genome-wide average from *RFmix*), and a source allele frequency given by the present-day Druze population of 36%. Even if we assume that the proportion of non-African haplotypes introduced is 60%, which is the observed value at the ADH1B*48His site (fig. 6*a*), the selection coefficient is still significantly higher than zero: *s*^′^ = 0.89% (0.66–1.1%, LR_100_). These estimates are likely conservative, given that we do see a signal of positive selection at ADH1B*48His in the Druze (though it is weaker than in Afroasiatic Africans; supplementary fig. S12 and table S2, Supplementary Material online), meaning that the allele frequency of ADH1B*48His was likely lower than the current observed value when gene flow into Africa occurred ∼2,000 years ago. To confirm this, we examined the frequency of ADH1B*48His in a data set of 73 ancient individuals dating to the bronze and iron ages (∼3,000–5,000 years ago) in the Southern Levant (Agranat-Tamir et al. 2020). We find that the ADH1B*48His allele is at 0% frequency in this data set, suggesting that the allele is at a higher frequency in the Levant today than it was when gene flow into Africa occurred. Again, these results are consistent either with selection starting slightly earlier in the Levant than Africa, or weaker selection in Levant, though our current data do not allow us to disentangle these possibilities.

Finally, we sought to understand whether the signal of positive selection we observe in the Ethiopian Semitic/Cushitic population is unusual compared with what we might expect across the genome, given this recent admixture. Recent admixture can introduce long novel haplotypes into a population that can be mistakenly interpreted as resulting from a selective sweep when using haplotype-based tests of selection (Walsh et al. 2020). To test whether this locus contains longer haplotypes than expected given the recent admixture, we compared the iHS value of the target ADH1B*48His to all background SNPs that satisfied three conditions: First, the background SNP had to have an allele with the same frequency as ADH1B*48His in the Druze, and second, this allele must have a 0% frequency in African populations outside of Northeast Africa. For this second consideration, we used the Nigerian Yoruba (YRI) from the 1KGP data set as a proxy. Third, the background SNP had to have a minor allele frequency >5% in the focal Ethiopian population. This resulted in a list of 1,410 background variants for comparison with our target SNP. We then examined the distribution of normalized iHS scores at these matched background SNPs in the focal Ethiopian Semitic/Cushitic population, and compared it against the iHS value observed for ADH1B*48His (supplementary fig. S13, Supplementary Material online). We find that ADH1B*48His is an outlier compared with this distribution, with an extreme iHS compared with the set of background SNPs (empirical *P* = 0.005; supplementary fig. S13*a*, Supplementary Material online). This result suggests that compared with all other SNPs at a similar frequency in the source population that could have introgressed into Northeast Africa, ADH1B*48His displays an usually strong signature of selection and is located on an unusually long haplotype. We also applied this method to iHS scores calculated in a pastoralist population (Beja) and a hunter-gatherer population (Weyto) who possess the ADH1B*48His allele. We find a slightly weaker signal in the pastoralists (*P* = 0.022; supplementary fig. S13b, Supplementary Material online), and no signal of selection in the hunter-gatherers (*P* = 0.21; supplementary fig. S13c, Supplementary Material online), further supporting the hypothesis that agriculture is the selective force acting on this variant.

## Discussion

In this study, we show that the ADH gene region, which includes the ADH1B*48His allele, has experienced recent positive selection in multiple agriculturalist populations from Ethiopia. This signal is within the top 0.5% of all SNPs genome-wide in both *F*_ST_ and haplotype-based tests of positive selection. In contrast, we show that geographically proximate hunter-gatherers and pastoralists from the same region do not display a signal of selection at this locus, even though they possess the ADH1B*48His allele and share genetic ancestry. This observation bolsters the argument that the emergence of agriculture has shaped patterns of selection at this locus. While other authors have suggested a relationship between selection at the ADH gene region and agriculture, our study is the first to examine this hypothesis in populations outside of East Asia. Further, we show that these ADH haplotypes likely introgressed from a Eurasian source into Ethiopia within the last ∼2,000 years and experienced positive selection post-admixture. Specifically, we conservatively estimate a post-admixture selection coefficient for ADH1B*48His of 1.12%. For comparison, a recent study estimated a selection coefficient for this allele in East Asians of ∼2%, starting ∼4,000 years ago (Mathieson 2020). Although our results do not pinpoint the exact mutational target of selection in the ADH gene region nor the ultimate cause for the apparent fitness advantage, we do show evidence that an agricultural subsistence strategy may be involved. This link between agriculture and the ADH gene region could have multiple possible explanations.

First, as other authors have noted, the emergence of Neolithic agriculture led to an associated increase in the availability of fermented foods and beverages in many cultures (McGovern et al. 2004). Fermentation not only allows for the production of alcoholic beverages, but is also used to preserve or enhance the nutritional properties of many foods. However, because of the addictive properties of ethanol and its damaging effects on multiple body systems (Polimanti and Gelernter 2018), genes involved in alcohol metabolism could become targets of selection in populations with a high prevalence or long history of these fermented products. For example, Peng et al. (2010) previously showed that the frequency of the ADH1B*48His allele in East Asians is higher in ethnic groups that adopted rice agriculture earlier. In the Ethiopian populations examined here, many traditional fermented beverages are produced and widely consumed, including *tella*, *tej*, *areki*, *borde*, and *shamita* (Tafere 2015). In addition, the national dish of Ethiopia is *injera*, a fermented flatbread for which many Ethiopians completely or partially depend on for a substantial portion of their diet. While these fermented products have a long history in Ethiopia, it is also possible that the selective force acting on ADH1B*48His and other ADH gene variants is unrelated to alcohol metabolism.

Although ADH1B*48His and other ADH gene mutations are best studied for their role in the alcohol metabolism pathway and their effects on alcohol drinking behaviors (Li, Zhao, et al. 2011; Bühler et al. 2015), many studies have examined their roles in other pathways and phenotypes. For example, the *ADH1B* enzyme is involved in the metabolism of multiple compounds other than ethanol, including fatty acids, acetone, glucose, retinol, serotonin, and norepinephrine, among many others (Polimanti and Gelernter 2018). Further, Mendelian randomization analyses have found strong associations between ADH1B*48His and cardiovascular phenotypes. Holmes et al. (2014) conducted a large-scale randomization analysis of 261,991 Europeans and found that carriers of the ADH1B*48His allele not only consumed less alcohol, but had significantly lower blood pressure, BMI, inflammatory biomarkers, and non-high-density lipoprotein cholesterol than noncarriers. Carriers of this allele also had a reduced risk of coronary heart disease. More recently, Polimanti et al. (2016) conducted a large-scale phenome-wide association study (pheWAS) for the ADH1B*48His allele for a range of physical and mental health traits. They found that the ADH1B*48His genotype was significantly associated with metabolic traits including BMI and dietary energy intake and found suggestive associations between this allele and lower pulse pressure and systolic blood pressure (Polimanti et al. 2016). Further, a large-scale study of a Japanese cohort (*n* = 135,974) found that the ADH1B*48His allele is significantly associated with “all-cause mortality,” with the derived allele conferring a survival advantage over the ancestral allele that is independent of alcohol consumption levels (Sakaue et al. 2020). *A* large-scale pheWAS study in African cohorts for ADH loci could help clarify some of the other possible phenotypes that selection may be acting on in these populations. Finally, a study in a Korean cohort identified a statistical association between the missense variant rs671 in the *ALDH2* gene, which also plays a role in ethanol metabolism, and reduced risk of tuberculosis (Park et al. 2014). This result suggests the intriguing possibility that genes involved in alcohol metabolism may be involved in susceptibility to environment-mediated infectious disease exposure, which could represent a strong selective force in Africa.

It is possible that selection might not be acting on ADH1B*48His, but on another nearby variant or on the synergistic effects of multiple variants on the same extended haplotype. One candidate in this regard is the nonsynonymous variant rs1573496 in the *ADH7* gene, ∼110 kb upstream of ADH1B*48His (table 1, fig. 3, supplementary fig. S3, Supplementary Material online). This variant shows a very similar allele frequency distribution to ADH1B*48His within Northeast Africa, and outside of Africa occurs at appreciable frequency only in the Levant (fig. 3*f*). This variant is in relatively high, but not complete, LD with ADH1B*48His in the focal Ethiopian Semitic/Cushitic population (*R*^2^ = 0.47, *D*′ = 0.83), suggesting that selection acting on this variant may be partially independent to that acting on ADH1B*48His. The *ADH7* gene is involved in the metabolism of ethanol into acetaldehyde, but is expressed in the epithelial tissues of the aerodigestive tract (Jairam and Edenberg 2014). In a study examining 3,876 cases and 5,278 controls, this *ADH7* variant was significantly protective against upper aerodigestive cancers (Hashibe et al. 2008). Moreover, this effect became more significant with increasing alcohol consumption, and was independent of the protective effect of ADH1B*48His. Because the cancer-protecting effects of this variant appear to be alcohol related, it is possible that selection may favor this allele in populations where consumption of fermented foods and beverages is historically high. However, if cancer strikes after reproductive age, this may not be the primary source of selection acting on this variant.

Another interesting candidate which could be a target of selection is a nonsynonymous variant at rs113337987 in the *MTTP* gene ∼300 kb upstream of *ADH1B*, which in fact has the highest iHS of any nonsynonymous variant in the ADH region in the Ethiopian Semitic/Cushitic population (table 1). Unlike some of the other discussed variants, this variant shows the highest global allele frequency in Ethiopia, has a lower allele frequency in the Levant, and is largely absent from all other global populations (fig. 3*g*–*i*). The MTTP protein is involved in the production of beta-lipoproteins, including low-density lipoproteins (LDLs) and very low-density lipoproteins (VLDLs). Accordingly, several studies have shown that mutations in the *MTTP* gene significantly associate with lower LDL levels and other metabolic traits (Ledmyr et al. 2002; Lundahl et al. 2006). *MTTP* also plays a role in the assembly of chylomicrons, which transport dietary lipids from the intestines to the bloodstream (Sharp et al. 1993). Many mutations in this gene are associated with abetalipoproteinaemia, a disorder characterized by an impaired ability to absorb dietary fats and fat-soluble vitamins (Welty 2014). Interestingly, ethanol has been shown to down-regulate the expression of *MTTP* in rats as well as human liver cells, perhaps due to a negative ethanol response element in the gene’s promoter region (Lin et al. 1997; Sugimoto et al. 2002). Mutations in the *MTTP* gene have also been shown to play a role in the development of nonalcoholic fatty liver disease (Hsiao et al. 2015; Gouda et al. 2017). Together, these studies suggest a possible functional link between the *MTTP* gene and ADH genes, which may influence selection signals at these loci. Future functional work will be needed to disentangle these different possibilities.

Our results indicate that these ADH gene region mutations may have been recent additions to the African genomic landscape. Our estimate of an admixture timing of ∼2,000–2,500 years ago from Eurasia into Ethiopia is similar to estimates by other authors. For example, Pagani et al. (2012) used the similar ROLOFF method (Moorjani et al. 2011) to estimate an admixture timing of ∼2,500–3,000 years ago between Ethiopian populations and non-Africans. Similarly, Pickrell et al. (2014) estimated an admixture timing of ∼2,500–3,500 years ago between Ethiopia and Eurasia. Our slightly more recent estimate may be due to the fact that simulation studies show that if admixture between populations is not a singular event, but rather occurs in multiple pulses, the ALDER methodology used here will recover an admixture date closest to the most recent pulse (Hodgson et al. 2014; Pugach et al. 2018). It is also possible that populations in Ethiopia have experienced non-African gene flow continuously over a longer period of time. Regardless, our results suggest that positive selection-only drove functional ADH variants to high frequency in populations practicing agriculture.

In sum, this study contributes significantly to our understanding of the evolutionary history of the ADH gene region in humans and local adaptation in Africa. We show that the ADH gene region has experienced positive selection in a set of populations from Ethiopia, and that this signal is correlated with an agricultural subsistence strategy. We further show that the haplotypes carrying putative functional ADH variants almost certainly originated from outside of Africa, and positive selection likely occurred post-admixture. Together, these analyses improve our understanding of the ways in which humans have adapted to different diets and environmental contexts through evolutionary time, and highlight some of the advantages of including indigenous African populations in studies of human adaptation.

## Materials and Methods

### Sample Acquisition and Genotyping

We obtained IRB approval for this project from the University of Pennsylvania, and we obtained written informed consent from all study participants. We obtained ethics approval from the following institutions: the National Institute of Medical Research in Dar es Salaam, Tanzania; COSTECH (the Tanzania Commission for Science and Technology); the Kenya Medical Research Institute, Nairobi; the University of Khartoum, Sudan; the Nigerian Institute for Research and Pharmacological Development, Nigeria; the Ministry of Health and National Committee of Ethics, Cameroon; the University of Addis Ababa, Ethiopia; the Federal Democratic Republic of Ethiopia Ministry of Science and Technology National Health Research Ethics Review Committee, Ethiopia. DNA was extracted from whole blood and genotypes for a total of 1,071 individuals were generated. The 541 individuals from Ethiopia were genotyped on the Illumina Omni5 Genotyping array (∼4.2 million SNPs; Crawford et al. 2017; dbGAP accession phs001396.v1.p1), while the remaining 530 African individuals from outside of Ethiopia were genotyped on the Illumina 1M-Duo bead array (∼1 million SNPs; Scheinfeldt et al. 2019; dbGAP accession phs001780.v1.p1; supplementary table S1, Supplementary Material online). We pre-phased both data sets with *SHAPEIT2* (Delaneau et al. 2012) using haplotypes from the 1KGP database (Auton et al. 2015) as a reference panel, and then imputed each data set separately using a reference panel of 180 diverse African whole genomes (Fan et al., unpublished) combined with global data from the 1KGP, using the program *minimac3* (Das et al. 2016). For the combined imputation reference panel, we took the union of variants between the 180 African whole-genome sequence data set and the 1KGP. In this way, variants specific to one data set but not the other were retained in the imputation reference panel. We then filtered both imputed data sets to only include high-quality SNPs with an *R*^2^ imputation score >0.7, and merged the intersection of the two data sets together using *bcftools*, resulting in a genome-wide data set of 19,902,653 SNPs. SNPs that were not present in the imputation reference panels and could not be imputed were discarded. Depending on the analysis performed (see below), we merged this “Africa-diversity” data set with global populations from the 1KGP data set, populations from the HGDP genotyped at 627,421 SNPs (Lazaridis et al. 2014), or with populations from the HGDP data set with high-coverage full-genome sequences (Bergström et al. 2020).

To verify imputation accuracy at the ADH region, we compared genotype concordance in a 2-Mb region centered on the ADH genes between 135 samples for which we had imputed genotype array data as well as full-genome sequencing data (Fan et al. unpublished). We find that genotype concordance in this region is 99.7%. The genotype concordance rate between these 135 samples for the three focal SNPs of interest (ADH1B*48*His*, rs1573496 of *ADH7*, and rs113337987 of *MTTP*) was also high (100%, 96.2%, and 100%, respectively). This high genotype concordance rate is also supported by the fact that these three variants have high estimated imputation accuracy *R*^2^ scores from *minimac3* in the full imputed data set (*R*^2^ = 0.89, 0.96, and 1 for the 3 variants, respectively).

### Estimation of Genetic Structure

To estimate population structure, we performed clustering analysis with the program *ADMIXTURE* (Alexander et al. 2009) and PCA using the *smartpca* program from the *EIGENSOFT* package (Price et al. 2006). To do this, we merged the Africa-diversity data set with the 1KGP and the high-coverage whole-genome sequence HGDP data set (Bergström et al. 2020), retaining only sites with an imputation *R*^2^ quality score >0.99. We further filtered out sites with a minor allele frequency <1% and removed all A/T and C/G SNPs. We used *PLINK* (v. 1.9) to calculate pairwise LD among sites within 100 kb windows, and excluded sites with pairwise *R*^2^ > 0.2 (Purcell et al. 2007), resulting in a data set of 234,157 SNPs for *ADMIXTURE* and PCA (fig. 1*b*and*c*). We present *ADMIXTURE* results for *K-*values of 9–11, with *K* = 11 having the lowest cross-validation error (fig. 1*b*and*c*). We ran *smartpca* using default parameters.

### Tests of Positive Selection

We used the program *selscan* to calculate the iHS genome-wide and to measure EHH signals at select variants (Szpiech and Hernandez 2014). To generate a fine-scale genetic map for use in these analyses, we used recombination maps from the HapMap phase II release (Frazer et al. 2007), and interpolated genetic positions for all variants in the African data set using the “approx” function in R (v.3.6.1; R Core Team 2019). We restricted our iHS calculations to sites with a minor allele frequency >5%, and where a confident ancestral allele could be established according to data released by the 1KGP. For haplotype-based selection analyses utilizing the pooled Ethiopian Semitic/Cushitic population (*N* = 220), we used the imputed Ethiopian data without merging with other sub-Saharan African populations, and restricted the analyses to variants with an *R*^2^ imputation quality score >0.85. Once iHS scores were calculated for all variants, we binned variants into 1% derived allele frequency bins, and normalized iHS scores to have a mean of zero and variance of one relative to SNPs within the same frequency bin. We report the absolute value of this normalized iHS.

To calculate the *D_i_* statistic, we followed the method developed by Akey et al. (2010). For each SNP, we calculated $D_{i}=\underset{\;j\;\neq \;i}{\sum }\;\frac{F_{\mathrm{ST}}^{ij}-\;E[F_{\mathrm{ST}}^{ij}]}{sd[F_{\mathrm{ST}}^{ij}]}$, where $E[F_{\mathrm{ST}}^{ij}]$ is the mean *F*_ST_ across all loci between populations *i* and *j*, and $sd[F_{\mathrm{ST}}^{ij}]$ is the standard deviation of *F*_ST_ between populations *i* and *j*. We performed all pairwise *F*_ST_ calculations between populations using the *VCFtools* (v0.1.17) implementation of the Weir and Cockerham (1984) formula. We derived the *D_i_* statistic for all SNPs across the genome for the populations listed in supplementary table S1, Supplementary Material online. For all tests of selection, we pruned the data set so that no pairs of individuals were retained with first- or second-degree relationships, using the program *KING* (Manichaikul et al. 2010). We report *P*-values for top selection hits as empirical *P*-values, which correspond to the percentile of the empirical distribution in which the SNP is located. We further note that these empirical *P*-values are not corrected for multiple testing.

### Haplotype Networks

To construct global haplotype networks around variants in the ADH gene region, we first created a merged data set that included a subset of the African populations from the Africa-diversity data set, a subset of the populations from the 1KGP, and a subset of populations from the HGDP high-coverage whole-genome sequencing data set available from Bergström et al. (2020). We used the “LiftoverVcf” tool implemented in *GATK* (v4.1.3.0) to convert the HGDP data from hg38 coordinates to hg19 coordinates prior to merging (Van der Auwera et al. 2013), and removed all A/T and C/G SNPs as well as sites with a MAF < 5%. We extracted phased regions around the ADH gene region from this data set and constructed median joining haplotype networks in *PopART* (v1.7; Leigh and Bryant 2015).

### Local Ancestry Analysis and Estimation of Admixture Timing

We performed local ancestry inference using the program RFmix (v2; Maples et al. 2013). To do this, we first merged the Ethiopian data set with the high-coverage HGDP whole-genome sequencing data set, retaining only high-quality imputed variants with an *R*^2^ imputation score >0.85. We further removed all A/T and C/G SNPs and filtered out variants with a minor allele frequency <5% in the combined query and reference populations, resulting in a genome-wide data set of 4,580,511 SNPs. We used the Yoruba (*N* = 22), Druze (*N* = 39), and Dizi (*N* = 39) as reference populations, and the pooled Ethiopian Semitic/Cushitic group (*N* = 220) as the query population. We used the “reanalyze reference” option with three expectation-maximization iterations in RFmix and assigned 68 generations since admixture between the reference populations (see admixture timings from *ALDER*). Regions of the genome assigned Druze ancestry by RFmix were designated “non-African,” while regions assigned Yoruba or Dizi ancestry were designated “African.”

To estimate the timing of admixture between a Eurasian source and Northeast African populations, we used the program *ALDER* (Loh et al. 2013). We merged the Africa-diversity data set with individuals from the HGDP genotyped on the Human Origins array (Lazaridis et al. 2014) and the 1KGP, resulting in a genome-wide data set of 434,686 SNPs. We estimated admixture timings for each Northeast African Afroasiatic-speaking ethnic group separately. For each of these analyses, we used the Yoruba (YRI) from the 1KGP as an African reference and the Druze (Israel) as a non-African reference.

### Parametric Selection Coefficient Estimate

The classic deterministic frequency evolution equation under a model of constant additive selection s on the coded allele is *ν*(*ν*_0_, *T*, *s*) = *x*_0_*e*^*sT*^/(1 + *x*_0_*e*^*sT*^), where *ν*_0_ is the initial frequency, *ν* is the frequency after *T* generations, and *x*_0_ = *ν*_0_/(1 − *ν*_0_). If the observed number of coded alleles is *k* and the total number of chromosomes in the sample is *N*, then the likelihood of observing *k* selected alleles under a selection-only model is given by *P*(*k*|*N*, *ν*_0_, *T*, *s*) = *f*_bin_(*k*, *N*, *ν*(*ν*_0_, *T*, *s*)), where $f_{\mathrm{bin}}(k,N,x)\;=\;\left(\begin{array}{c} N\\ k \end{array}\right)x^{k}(1-x{)}^{N-k}$ is the binomial probability. By Bayes’ Theorem, the likelihood of the selection coefficient *s* given the data {*k*, *N*} and parameters {*ν*_0_, *T*} is *P*(*s*|*k*, *N*, *ν*_0_, *T*) ∝ *P*(*k*|*N*, *ν*_0_, *T*, *s*), where the factor of proportionality is ignored as it does not enter into the likelihood maximization, that is, we choose flat priors over *s* and *k*. The maximum likelihood estimate of *s* is given by maximizing *P*(*k*|*N*, *ν*_0_, *T*, *s*), treating *s* as the free parameter. This was done using the python *scipy* library (Virtanen et al. 2020), specifically the *scipy.optimize.minimize* function on the negative log-likelihood, using the Nelder–Mead algorithm with a convergence criteria of tol = 1e−15.

For estimating the selection coefficient post-admixture in the pooled Ethiopian Semitic/Cushitic group, we use the Druze as a proxy source population. From this, we estimate the initial frequency of the ADH1B*48His allele on non-African haplotypes as *ν*_source_ = 0.36. The genome-wide average proportion of non-African haplotypes introduced into the pooled Ethiopian population is *f*_nah_ = 0.5 (the genome-wide average from *RFmix*). The ADH1B*48His allele is largely absent in other sub-Saharan African populations, and so we assume that the only source of ADH1B*48His alleles in the pooled Ethiopian Semitic/Cushitic population is from admixture with a non-African source. Accordingly, we use *ν*_0_ ≈ *ν*_source_*f*_nah_ = 0.18. The pooled Ethiopian Semitic/Cushitic group has *N* = 440 chromosomes, and the observed number of ADH1B*48His alleles is *k* = 147 (33% allele frequency). However, the proportion of non-African haplotypes at this site is higher than the genome-wide average, with 264 out of 440 haplotypes inferred as non-African (*f*_nah_ = 0.6; fig. 6*a*). Using this observation for estimating the initial allele frequency gives *ν*_0_ ≈ 0.22. For all estimates, we report the likelihood ratio interval of *s* values around the maximum likelihood estimate $\hat{s}$ such that the likelihood is within a factor of 100 of the maximum likelihood value, that is, all values *s* such that $P(k|s)/P(k|\hat{s})\geq 1/100$.

## Supplementary Material

Supplementary data are available at *Molecular Biology and Evolution* online.

## Supplementary Material

msac183_Supplementary_Data

## Data Availability

The raw genotype data underlying this article are available in dbGaP at https://www.ncbi.nlm.nih.gov/gap/, and can be accessed with accession numbers phs001396.v1.p1 and phs001780.v1.p1.
