# Supplementary material for: Signatures of Convergent Evolution and Natural Selection at the Alcohol Dehydrogenase Gene Region are Correlated with Agriculture in Ethnically Diverse Africans
Source: Mol Biol Evol. 2022 Aug 26;39(10):msac183. doi: 10.1093/molbev/msac183 (PMC9547508; doi:10.1093/molbev/msac183)
Supplement: msac183_Supplementary_Data [file msac183_supplementary_data.pdf]

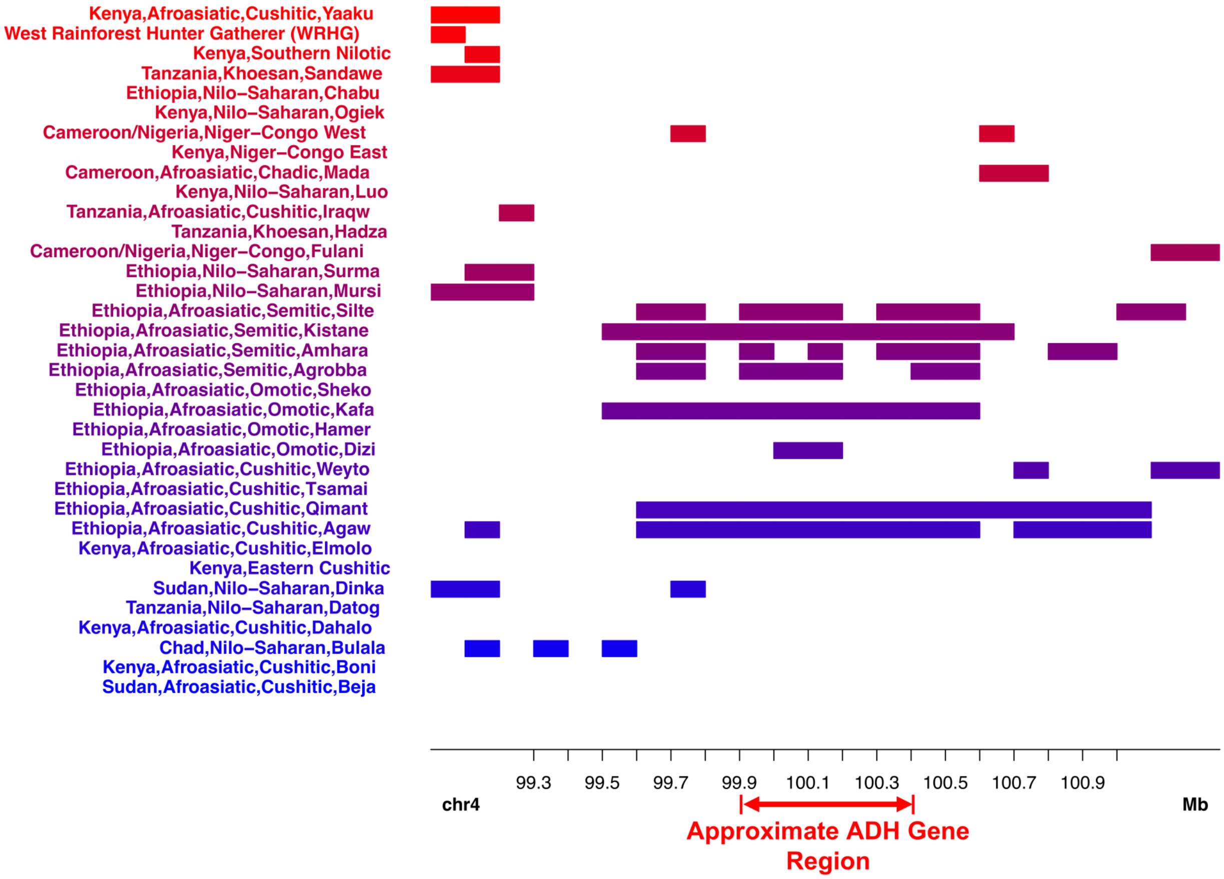

Fig. S1. Signature of elevated integrated haplotype score (iHS) across the ADH gene region in sub-Saharan Africans. Following Voight et al. (2006), we split the genome into non-overlapping 100kb windows, and for each population, we identified windows in the top 5% of the empirical distribution for the proportion of SNPs with extreme iHS scores (|standardized iHS > 2|). If a 100kb window was in the top 5% in this analysis, we colored that window in the plot. These windows in the ADH region are confined to Ethiopian Afroasiatic-speaking populations.

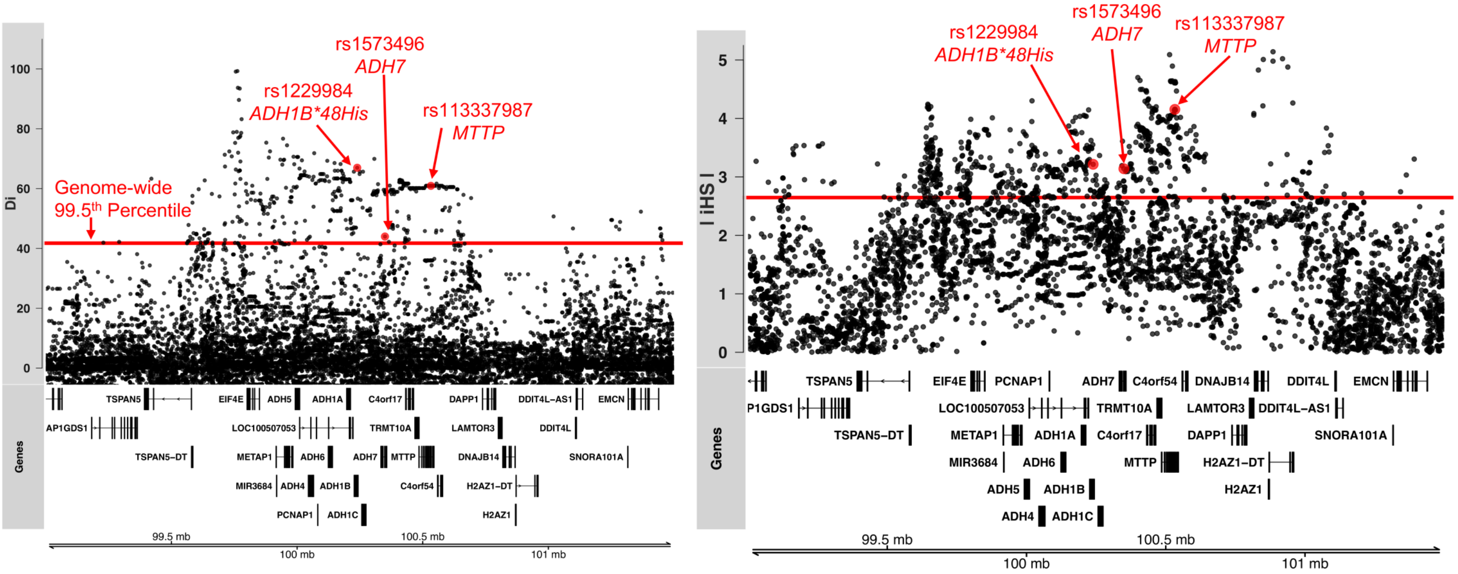

Fig. S2. Signatures of positive selection across the ADH gene region in the pooled Ethiopian Semitic/Cushitic population when filtering out all individuals with up to 3rd degree relationships (n=209). (**a**) D_i statistic and (**b**) iHS scores show a ~1.5 Mb region of elevated scores across the ADH region. Red line denotes genome-wide empirical 99.5th percentile for each statistic, and nonsynonymous mutations discussed in the main text are highlighted red.

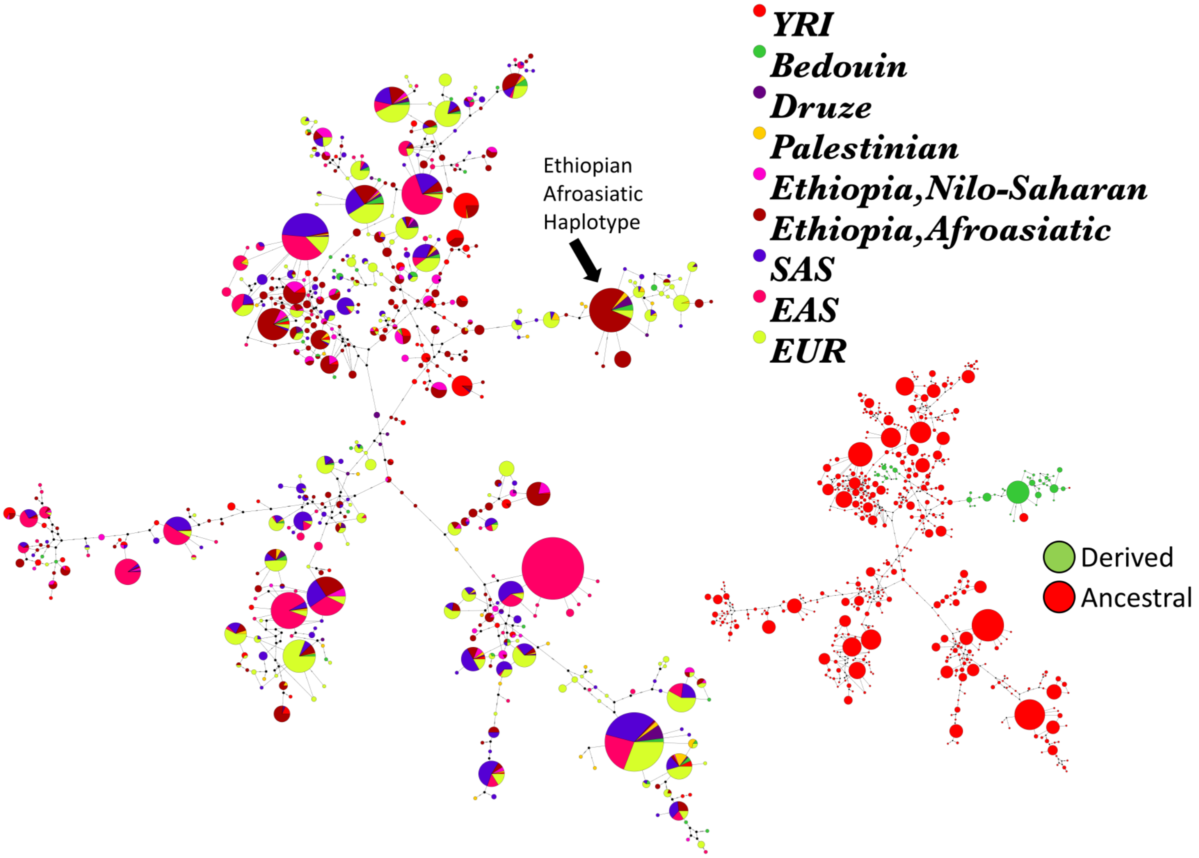

Fig. S3. 50kb median joining haplotype network around non-synonymous *ADH7* gene variant rs1573496 (112 SNPs). Larger network shows haplotypes colored by population, while smaller haplotype network is identical except that haplotypes are colored according to whether they carry the ancestral (red) or derived allele (green) at this locus. The Ethiopian Afroasiatic haplotype (dark red) carrying the derived allele is identical to a haplotype found in the Levant (Druze, Palestinian, Bedouin) and Europe (EUR), suggesting identity by descent.

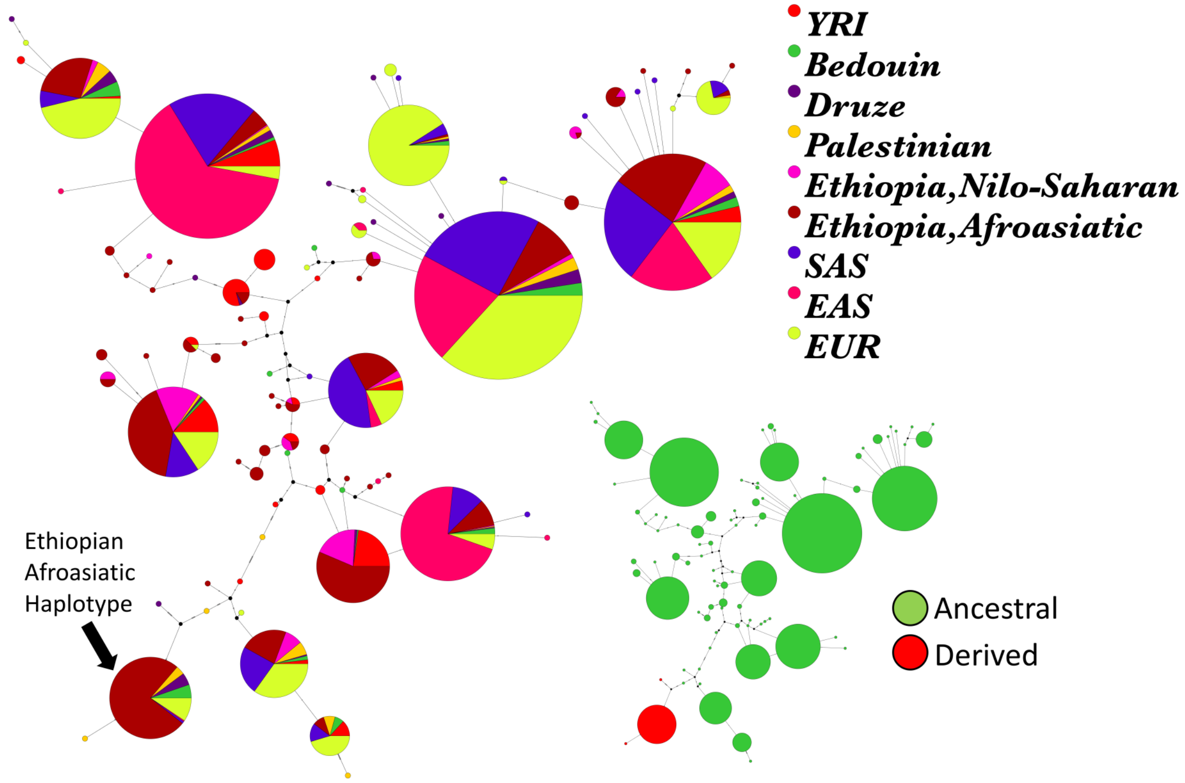

Fig. S4. 50kb median joining haplotype network around non-synonymous *MTTP* gene variant rs113337987 (102 SNPs). Larger network shows haplotypes colored by population, while smaller haplotype network is identical except that haplotypes are colored according to whether they carry the ancestral (green) or derived allele (red) at this locus. The Ethiopian Afroasiatic haplotype (dark red) carrying the derived allele is identical to a haplotype found in the Levant (Druze, Palestinian, Bedouin) and Europe (EUR), suggesting identity by descent.

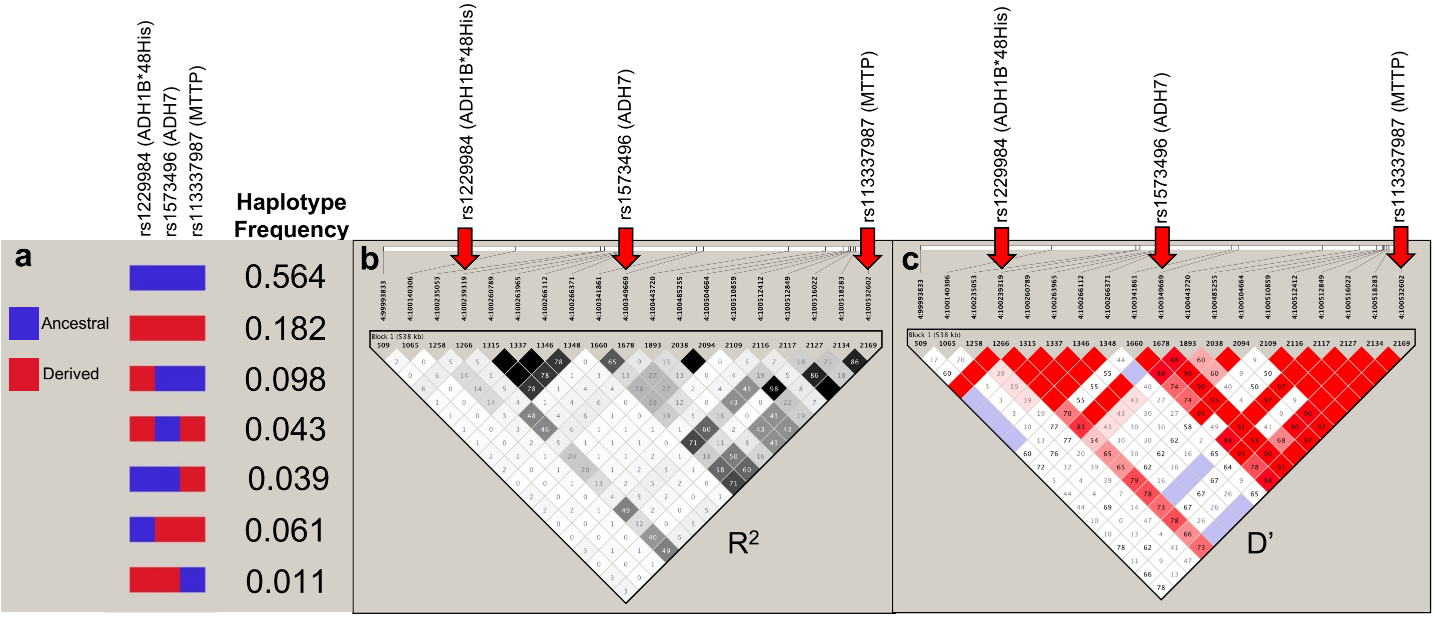

Fig. S5. Haplotype frequencies and patterns of linkage disequilibrium at the ADH gene region in the pooled Ethiopian Semitic/Cushitic population. (**a**) Haplotype frequencies at the three nonsynonymous variants showing the strongest positive selection signals. (**b**) Pairwise linkage disequilibrium (R^2) among all variants listed in Table 1. Colors correspond to R^2 value (black = 1, white = 0). (**c**) Pairwise linkage disequilibrium (D') among all variants listed in Table 1. Colors represent standard scheme in Haploview software and numbers in cells denote D'. Genetic positions are in the format 'chr:position' (hg19).

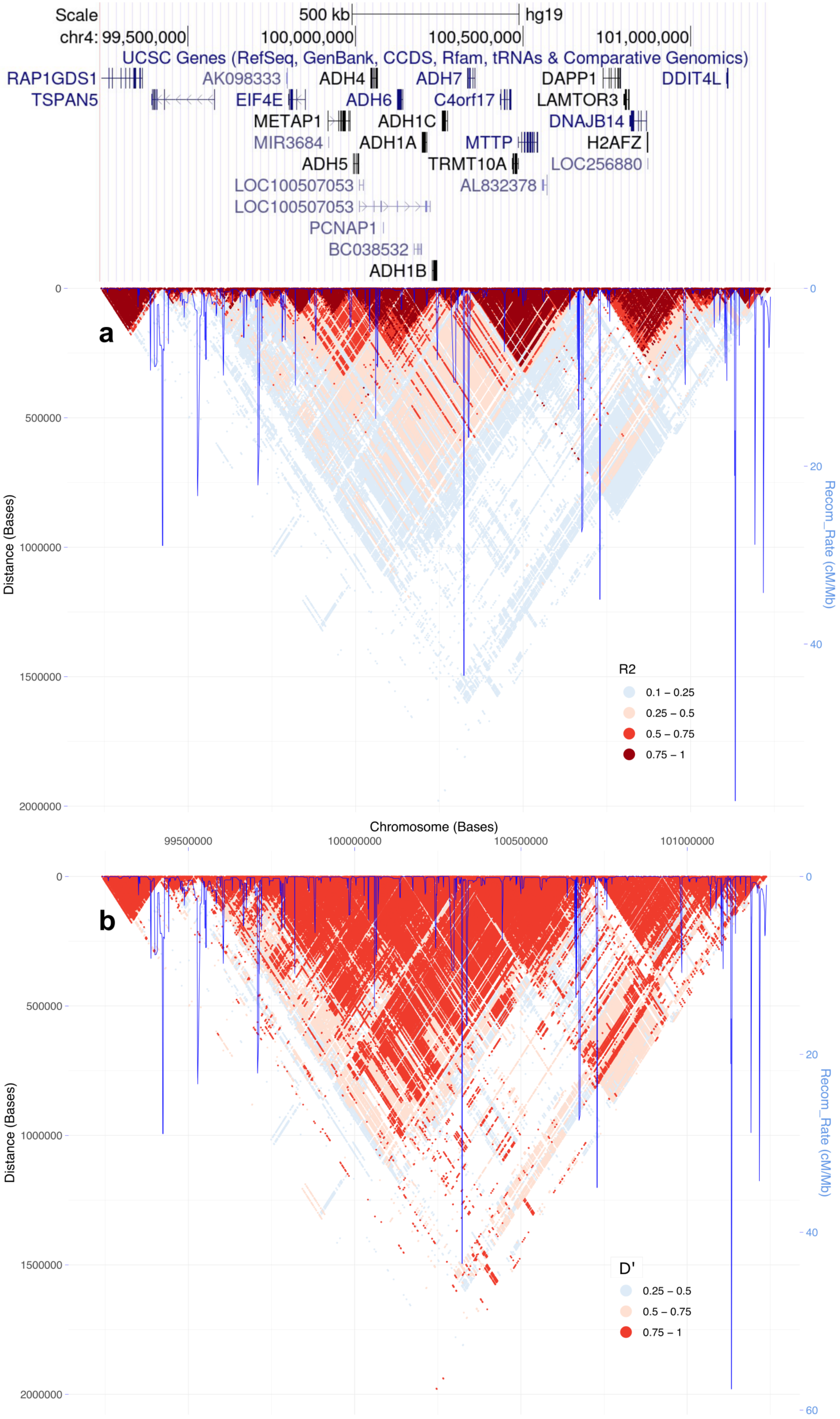

Fig. S6. Patterns of linkage disequilibrium (LD) across the ADH gene region in pooled Ethiopian Semitic/Cushitic population. (**a**) R^2 across the ADH region. (**b)** D prime across the ADH region. Recombination rate shown in blue.

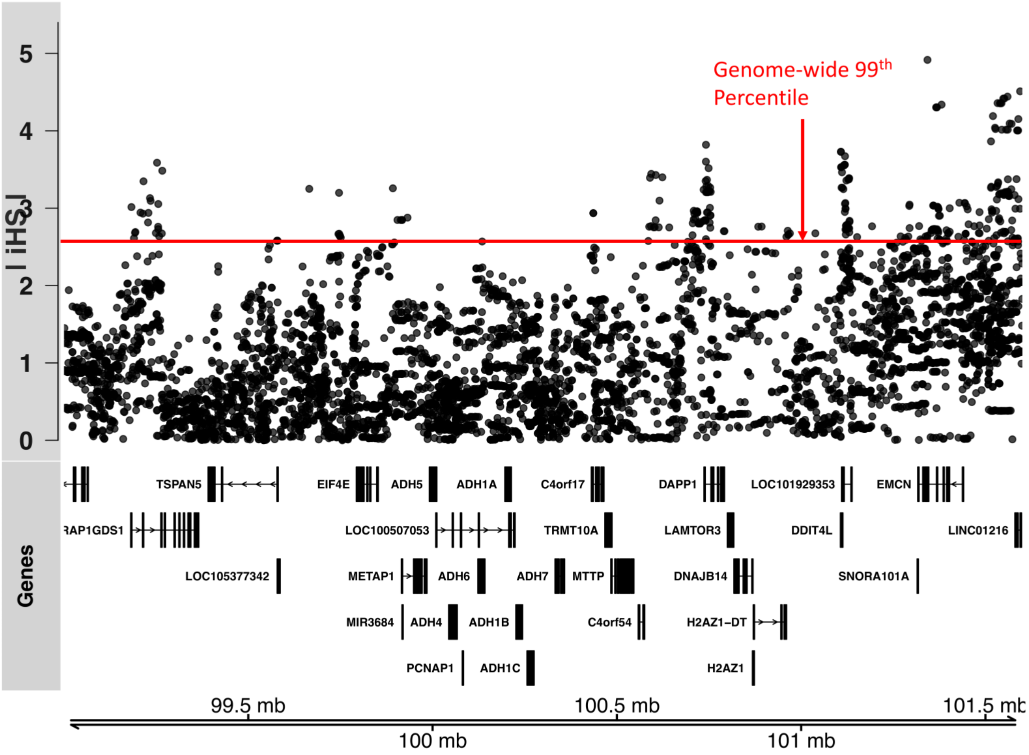

Fig. S7. Lack of signal of positive selection at the alcohol dehydrogenase gene region in the Weyto hunter-gatherers from Ethiopia. Integrated Haplotype Score (iHS) is shown in region surrounding alcohol dehydrogenase genes on chromosome 4. Very few SNPs in the ADH region show iHS scores above the genome-wide 99th percentile in this population.

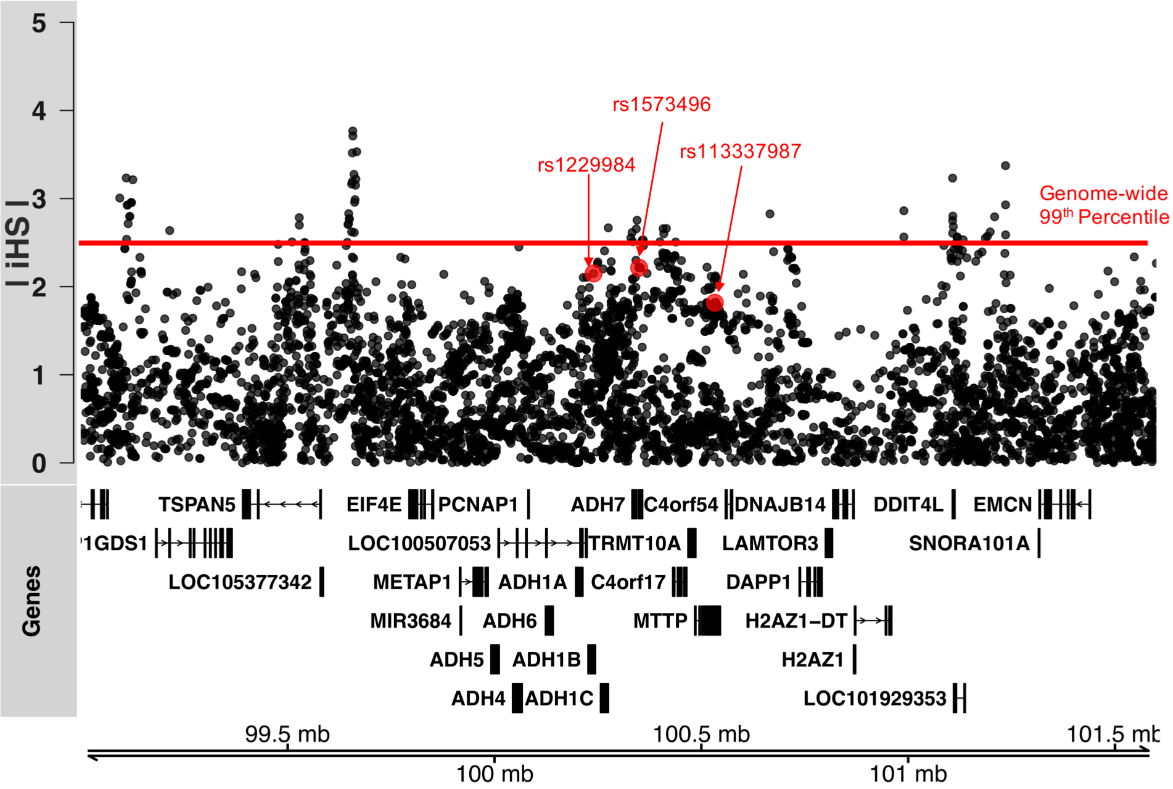

Fig. S8. Weaker signal of positive selection at the alcohol dehydrogenase gene region in the pastoralist Beja population from Kenya (contains Baniamer and Hadandawa ethnic groups). Integrated Haplotype Score (iHS) is shown in region surrounding alcohol dehydrogenase genes on chromosome 4. Very few SNPs in the ADH region show iHS scores above the genome-wide 99th percentile in this population.

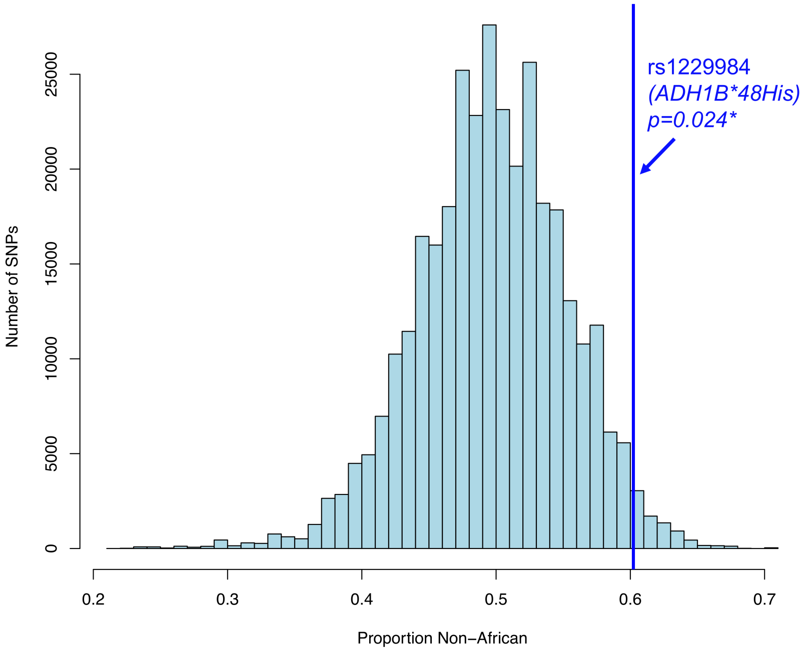

Fig. S9. Histogram showing the proportion of haplotypes in the pooled Ethiopian Semitic/Cushitic group inferred to be 'Non-African' at each SNP genome-wide. This analysis only contained variants with a MAF >= 30% and <= 35% in the focal Ethiopian Semitic/Cushitic population. Compared to the rest of the genome, the rs1229984 site (*ADH1B*48His*) (blue vertical line) is at the tail-end of the empirical distribution for the proportion of haplotypes in the population inferred to be non-African, suggesting positive selection post-admixture.

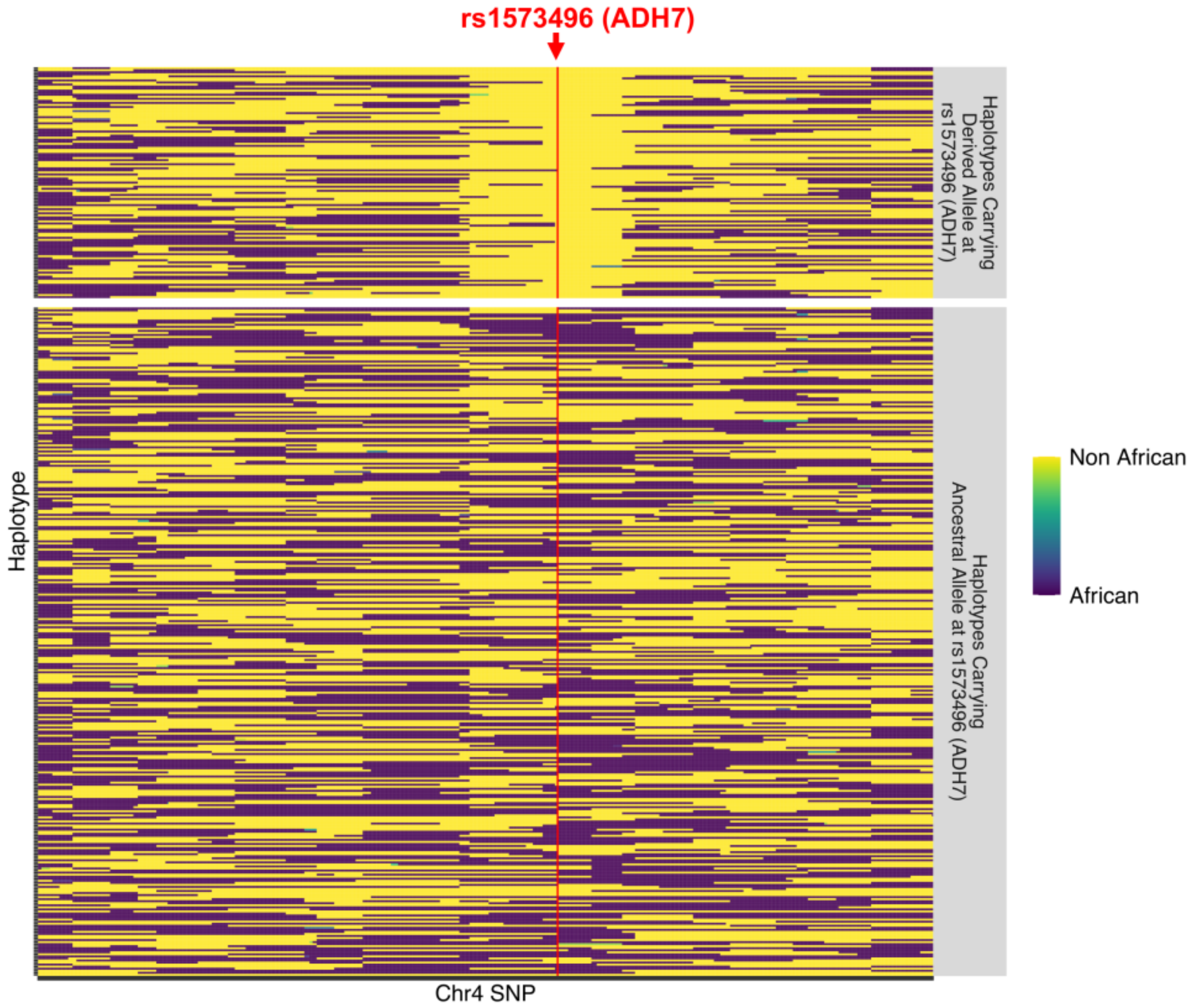

Fig. S10. RFmix local ancestry results showing enrichment in non-African ancestry at nonsynonymous SNP rs1573496 (*ADH7* gene). Each row is a haplotype, and the color of the haplotype represents African (purple) or non-African (yellow) ancestry inferred at each position along the haplotype. Haplotypes carrying the derived allele at rs1573496 are almost entirely inferred to be non-African in origin at this region.

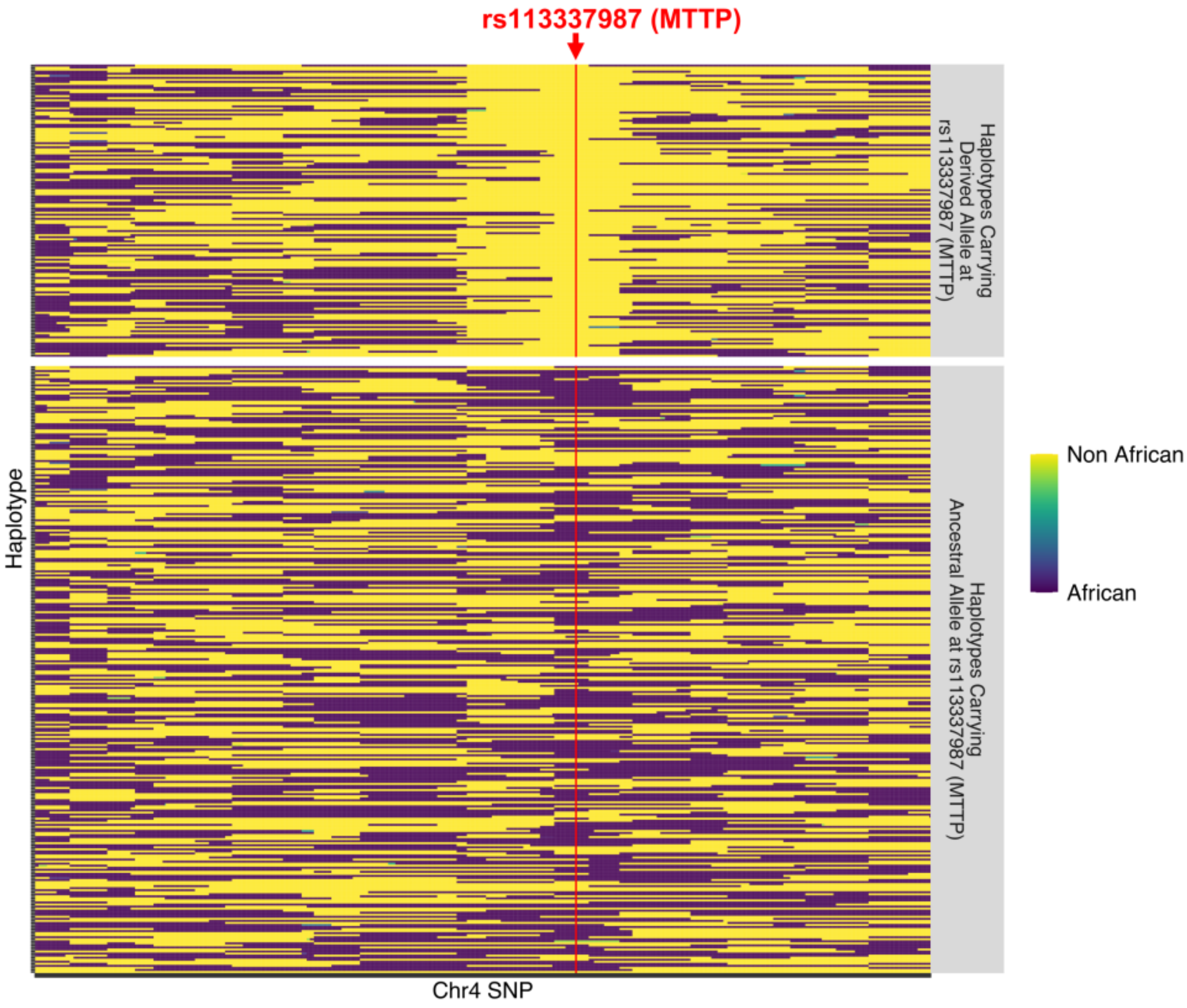

Fig. S11. RFmix local ancestry results showing enrichment in non-African ancestry at nonsynonymous SNP rs113337987 (MTTP gene). Each row is a haplotype, and the color of the haplotype represents African (purple) or non-African (yellow) ancestry inferred at each position along the haplotype. Haplotypes carrying the derived allele at rs113337987 are almost entirely inferred to be non-African in origin at this region.

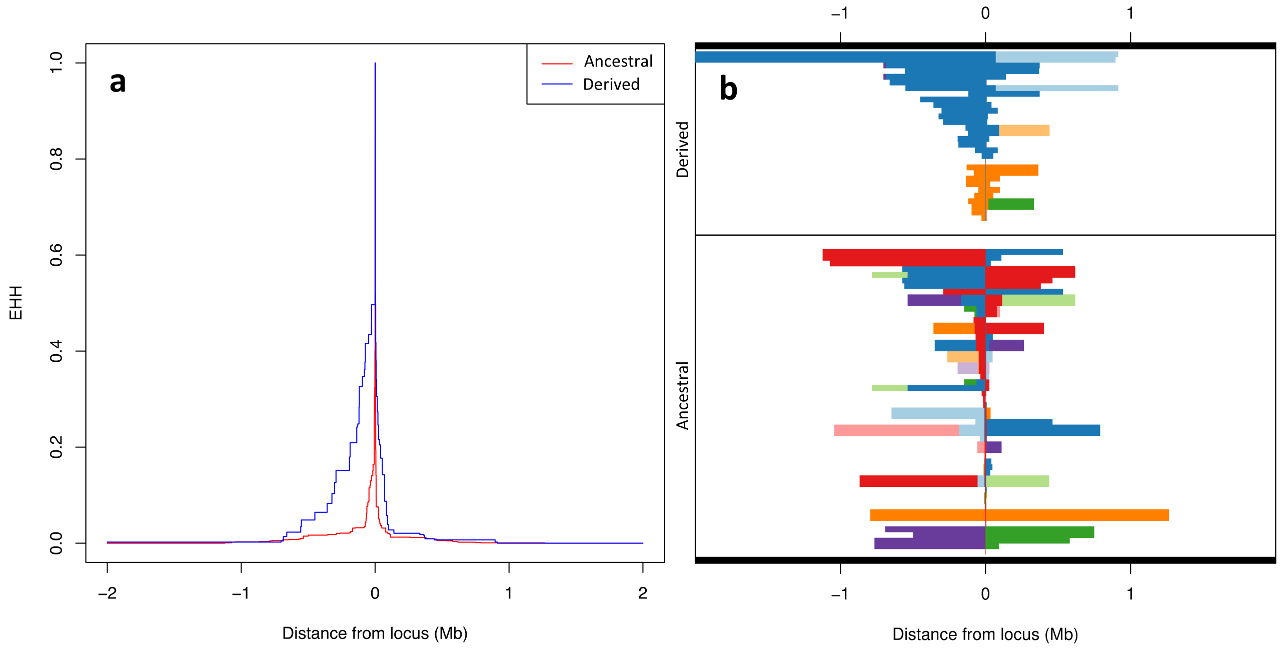

Fig. S12. Signals of Positive Selection at ADH1B*48*His* in the Druze (Israel) population. (**a**) Plot of extended haplotype homozygosity (EHH) centered on rs1229984 (*ADH1B*48His)*. (**b**) Plot of haplotype structure for chromosomes carrying the derived and ancestral alleles at rs1229984. Each row is a haplotype, and for each site, adjacent haplotypes of the same color are identical in sequence between that site and the central (selected) site.

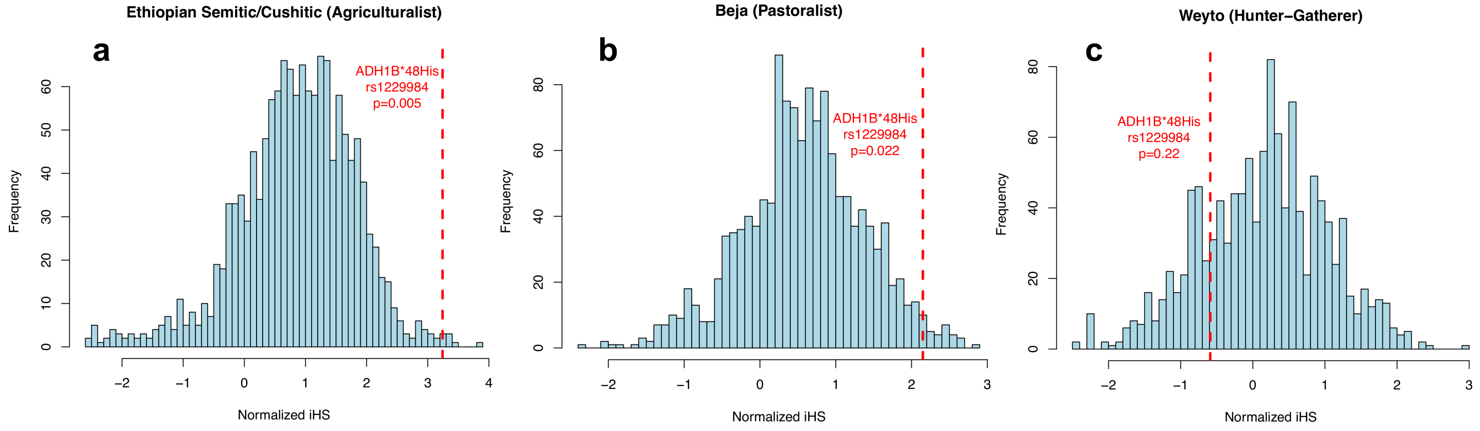

Fig. S13. Normalized iHS value of *ADH1B*48His* (red dashed line) compared against a background set of frequency matched SNPs. Background SNPs have the same allele frequency as ADH1B*48*His* in the Druze (introgression source), and a 0% frequency in the Nigerian Yoruba (YRI). ADH1B*48*His* is an iHS outlier in the pooled Ethiopian Semitic/Cushitic agriculturalists **(a)**, a more moderate outlier in the Beja pastoralists **(b)**, and not significantly different from background levels in the Weyto hunter-gatherers **(c)**.

Population Label	Country	Ethnic Group Composition	Language Family	Subsistence	Data Source
Ethiopia Semitic/Cushitic*<br>(Total N = 220)	Ethiopia	Agaw (N=68)<br>Amhara (N=40)<br>Qimant (N=34)<br>Kistane (N=28)<br>Argobba (N=23)<br>Silte (N=27)	Afroasiatic –<br>Semitic/Cushitic	Agriculturalist/<br>Agropastoralist	Crawford et al. 2017
Ethiopia Omotic*<br>(Total N = 89)	Ethiopia	Dizi (N=31)<br>Hamer (N=30)<br>Sheko (N=28)	Afroasiatic - Omotic	Agriculturalist/<br>Agropastoralist/<br>Pastoralist	Crawford et al. 2017
Ethiopia Nilo-Saharan*<br>(Total N = 42)	Ethiopia	Mursi (N=28)<br>Surma (N=14)	Nilo-Saharan	Pastoralist	Crawford et al. 2017
Weyto	Ethiopia	Weyto (N=35)	Afroasiatic - Cushitic	Hunter Gatherer	Crawford et al. 2017
Chabu*	Ethiopia	Chabu (N = 17)	Nilo-Saharan	Hunter Gatherer	Crawford et al. 2017
Tsamai	Ethiopia	Tsamai (N=34)	Afroasiatic – Cushitic	Agropastoralist	Crawford et al. 2019
Kafa	Ethiopia	Kafa (N=38)	Afroasiatic - Omotic	Agriculturalist	Crawford et al. 2019
Beja*<br>(Total N = 25)	Sudan	Baniamer (N=10)<br>Hadandawa (N=15)	Afroasiatic - Cushitic	Pastoralist	Scheinfeldt et al. 2019
Dinka*	Sudan	Dinka (N = 12)	Nilo-Saharan	Pastoralist	Scheinfeldt et al. 2019
Boni*	Kenya	Boni (N = 20)	Afroasiatic - Cushitic	Hunter Gatherer	Scheinfeldt et al. 2019
Dahalo*	Kenya	Dahala (N = 14)	Afroasiatic - Cushitic	Hunter Gatherer	Scheinfeldt et al. 2019
Eastern Cushitic*<br>(Total N = 30)	Kenya	Gabrah (N = 9)<br>Gurreh (N=7)<br>Rendille (N=14)	Afroasiatic - Cushitic	Pastoralist	Scheinfeldt et al. 2019
Elmolo*	Kenya	Elmolo (N = 13)	Afroasiatic - Cushitic	Hunter Gatherer	Scheinfeldt et al. 2019
Niger-Congo-East*<br>(Total N = 25)	Kenya	Pare (N=14)<br>Taita (N=9)<br>Taveta (N=2)	Niger-Congo	Agropastoralist	Scheinfeldt et al. 2019
Luo*	Kenya	Luo (N = 32)	Nilo-Saharan	Pastoralist	Scheinfeldt et al. 2019
Ogiek*	Kenya	Ogiek (N = 17)	Nilo-Saharan	Hunter Gatherer	Scheinfeldt et al. 2019
Southern Nilotic*<br>(Total N = 21)	Kenya	Pokot (N = 5)<br>Sengwer (N=16)	Nilo-Saharan	Agriculturalist	Scheinfeldt et al. 2019
Yaaku*	Kenya	Yaaku (N = 15)	Afroasiatic - Cushitic	Hunter Gatherer	Scheinfeldt et al. 2019
Datog*	Tanzania	Datog (N = 18)	Nilo-Saharan	Agropastoralist	Scheinfeldt et al. 2019
Hadza*	Tanzania	Hadzabe (N = 26)	Khoisan	Hunter Gatherer	Scheinfeldt et al. 2019
Iraqw*	Tanzania	Iraqw (N = 23)	Afroasiatic - Cushitic	Agropastoralist	Scheinfeldt et al. 2019
Sandawe*	Tanzania	Sandawe (N = 29)	Khoisan	Hunter Gatherer	Scheinfeldt et al. 2019
Mada*	Cameroon	Mada (N = 19)	Afroasiatic	Agriculturalist	Scheinfeldt et al. 2019
West Rainforest Hunter Gatherer (WRHG)*<br>(Total N = 67)	Cameroon	Baka (N = 25)<br>Bakola (N=29)<br>Bedzan (N=13)	Niger-Congo	Hunter Gatherer	Scheinfeldt et al. 2019
Niger-Congo-West*<br>(Total N = 70)	Cameroon/Nigeria	Lemande (N = 19)<br>Ngumba (N=20)<br>Tikar South (N=19)<br>Yoruba (N=12)	Niger-Congo	Agriculturalist	Scheinfeldt et al. 2019
Fulani*<br>(Total N = 40)	Cameroon/Nigeria	Fulani Cameroon (N = 16)<br>Fulani Nigeria (N=8)<br>Mbororo Fulani Cameroon (N=16)	Niger-Congo	Pastoralist	Scheinfeldt et al. 2019
Bulala*	Chad	Bulala (N = 12)	Nilo-Saharan	Agropastoralist	Scheinfeldt et al. 2019

Table S1. Population groupings and ethnic group composition used for genome-wide scans of positive selection. Populations marked with an asterisk were included in the D_i calculations.

Chr	Position (hg19)	| iHS | (pval)	Ancestral	Derived	Gene	Function	rsID
4	100532602	2.01953 (0.04471436)	G	A	MTTP	nonsynonymous SNV	rs113337987
4	100239319	2.07323 (0.03957207)	C	T	ADH1B	nonsynonymous SNV	rs1229984
4	100349669	2.87947 (0.005451212)	C	G	ADH7	nonsynonymous SNV	rs1573496

Table S2. Integrated haplotype scoress (iHS) for 3 nonysnonymous variants in the Druze population from Israel. iHS was calculated genome-wide using high coverage HGDP data (Bergström, et al. 2019). iHS was normalized based on derived allele frequency, and the absolute value and empirical p values are shown.
